# Biomimetic synthesis and HPLC–ECD analysis of the isomers of dracocephins A and B

**DOI:** 10.3762/bjoc.12.247

**Published:** 2016-11-24

**Authors:** Viktor Ilkei, András Spaits, Anita Prechl, Áron Szigetvári, Zoltán Béni, Miklós Dékány, Csaba Szántay, Judit Müller, Árpád Könczöl, Ádám Szappanos, Attila Mándi, Sándor Antus, Ana Martins, Attila Hunyadi, György Tibor Balogh, György Kalaus (†), Hedvig Bölcskei, László Hazai, Tibor Kurtán

**Affiliations:** 1 Department of Organic Chemistry and Technology, Budapest University of Technology and Economics, Szt. Gellért tér 4, H-1111 Budapest, Hungary; 2 Gedeon Richter Plc., Gyömrői út 19-21, H-1103 Budapest, Hungary; 3 Department of Organic Chemistry, University of Debrecen, P. O. Box 400, H-4002 Debrecen, Hungary; 4 Department of Medical Microbiology and Immunobiology, University of Szeged, Dóm tér 9, H-6720 Szeged, Hungary; 5 Permanent address: Synthetic Systems Biology Unit, Institute of Biochemistry, Biological Research Centre, Temesvári krt. 62, H-6726 Szeged, Hungary; 6 Institute of Pharmacognosy, Faculty of Pharmacy, University of Szeged, Eötvös utca 6, H-6720 Szeged, Hungary

**Keywords:** absolute configuration, *Dracocephalum rupestre*, dracocephins A–B, ECD calculation, flavonoid alkaloids, HPLC–ECD

## Abstract

Starting from racemic naringenin ((±)-**1**), a mixture of dracocephin A stereoisomers 6-(2”-pyrrolidinone-5”-yl)naringenin (±)-**2a–d** and its regioisomer, dracocephin B 8-(2”-pyrrolidinone-5”-yl)naringenin (±)-**3a–d** originally isolated from *Dracocephalum rupestre*, have been synthesized in a one-pot reaction. The separation of **2a–d** and **3a–d** was achieved by preparative HPLC. The four stereoisomers of each natural product were separated by analytical chiral HPLC and their absolute configuration was studied by the combination of HPLC–ECD measurements and TDDFT–ECD calculations. The synthesized flavonoid alkaloids were further characterized by physicochemical and in vitro pharmacological studies.

## Introduction

**Figure 1:**
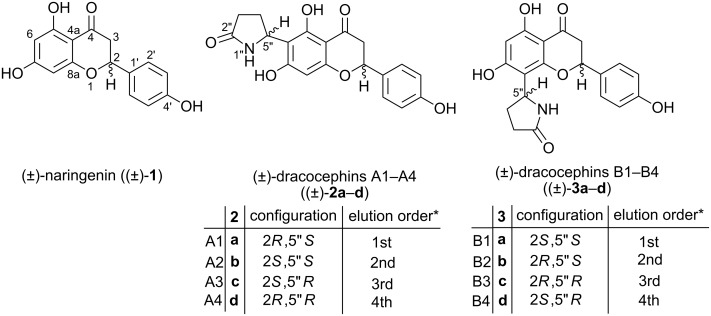
Structures of (±)-naringenin, (±)-dracocephins A1–A4 and B1–B4 with the indication of the absolute configuration and elution orders for separation on Chiralpak AS-H* column [2]. The absolute configurations are presented as proposed by Ren et al. [2].

Flavonoid alkaloids are a small subgroup of flavonoids possessing a five- or six-membered nitrogen heterocycle attached to C-6 or C-8 of ring A of the flavonoid moiety. They have been reported to exhibit a wide range of pharmacological activities [1]. Dracocephins A and B were isolated as a mixture of four stereoisomers by Ren et al. in 2008 from *Dracocephalum rupestre* [2], which is an herb widely distributed throughout western China and used in folk medicine for the treatment of various conditions including cold, cough, icterohepatitis and laryngalgia. Dracocephins A (±)-**2a–d** and B (±)-**3a–d** have been found to be conjugates of racemic naringenin ((±)-**1**) with pyrrolidine-2-one with C-6–C-5” and C-8–C-5” linkage, respectively. Due to the two chirality centers, four possible stereoisomers exist for each regioisomer, and accordingly, the isolated substances were verified to be 1:1 mixtures of two diastereoisomeric racemates (**2a**, **2c**/**2b**, **2d**; **3a**, **3c**/**3b**, **3d**) by HPLC–ECD analysis (Figure 1).

**Scheme 1:**
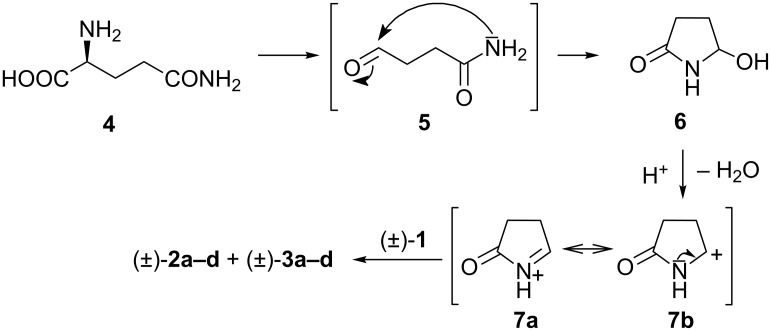
Proposed biosynthetic route to dracocephins A and B.

The planar structure and absolute configuration of the first-eluted stereoisomer of dracocephins A (±)-**2a–d** was determined by single-crystal X-ray diffraction analysis as (2*R*,5”*S*)-**2a** [2]. The biosynthesis of these flavonoid–pyrrolidone conjugates is proposed by Leete [3] and Tanaka et al. [4] to proceed via an acylaminocarbinol intermediate, which presumably arises through the Strecker-degradation of the corresponding amino acids, which is L-glutamine (**4**) in the case of dracocephins A and B as shown in Scheme 1.

The spontaneous cyclization of the Strecker aldehyde **5** yields the acylaminocarbinol intermediate **6**, which readily loses water on protonation, resulting in the *N*-acyliminium ion **7a**/**b**, a strong electrophilic reagent.

## Results and Discussion

The aim of our work was to synthesize the natural flavonoid alkaloids **2a**–**d** and **3a**–**d** in a biomimetic scheme in order to devise an efficient route to these natural products for structure–activity relationship studies.

**Scheme 2:**
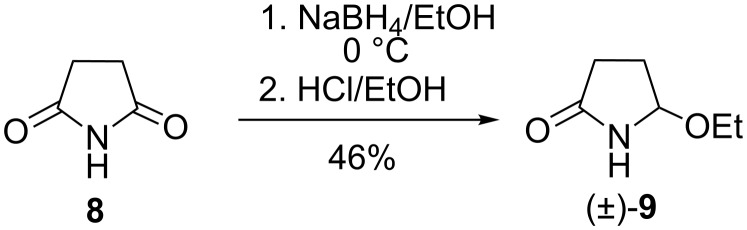
Synthesis of (±)-5-ethoxypyrrolidine-2-one ((±)-**9**).

First the *N*-acylaminocarbinol reagent was prepared in the form of racemic 5-ethoxypyrrolidine-2-one ((±)-**9**) by the partial reduction of succinimide (**8**) with sodium borohydride at 0 °C, as described in the literature [5] (Scheme 2).

**Scheme 3:**
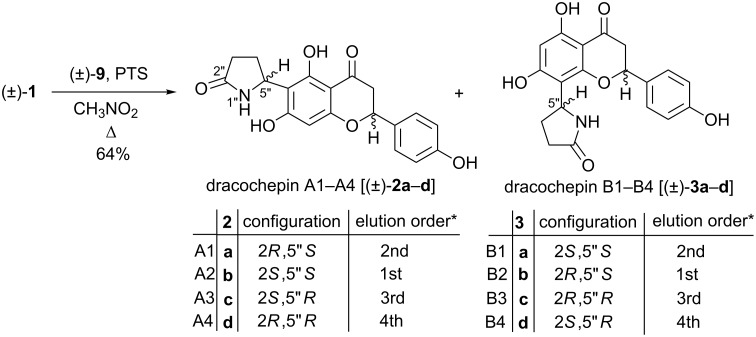
Synthesis of (±)-dracocephins A and B (±)-**2a**–**d** and (±)-**3a–d** and the elution order of stereoisomers on a Chiralpak IC* column with the eluent hexane/MeCN/TFA 97:3:0.1. Absolute configurations and the names of stereoisomers are shown as proposed by Ren et al. [2].

In the next step, dracocephins A (±)-**2a**–**d** and B ((±)-**3a**–**d**) were prepared by reacting racemic naringenin ((±)-**1**) with the *N*-acylaminocarbinol ether (±)-**9** in the presence of a catalytic amount of *p*-toluenesulfonic acid (PTS) in nitromethane at 101 °C for 4 h as shown in Scheme 3.

The product was purified by column chromatography resulting in a mixture of dracocephins A and B as a white crystalline solid in 64% yield. Although their mixture could not be separated on silica gel by column chromatography, the two isomeric products could be separated by preparative HPLC to result in regioisomers (±)-**2a**–**d** and (±)-**3a**–**d** as crystalline powders in a ratio of 43:57, respectively. These data clearly showed that the reaction took place without notable regioselectivity, which was in agreement with the observed 58:42 ratio of natural dracocephins A and B [2].

The regioisomeric dracocephins A and B were identified by NMR spectroscopy. The CD_3_OD solution of compounds (±)-**2a–d** showed a cross-peak between the singlet of H-8 and the multiplet of H-2’ in the 2D NOESY spectrum, which corroborated the structure of dracocephins A. Moreover, compounds (±)-**3a**–**d** showed 2D NOESY cross peaks between multiplets of the pyrrolidine ring and H-2’, corresponding to dracocephins B. Aliquots of sample (±)-**2a**–**d** and sample (±)-**3a**–**d** were dissolved in DMSO-*d*_6_ in order to allow comparison with literature data. The ^1^H chemical shifts of the herein synthesized dracocephins A and B showed good agreement with those of the isolated flavonoid alkaloids [2]. Similarly to the natural route, no diastereoselectivity occurred in this sequence, as proven by the separation of the stereoisomers by chiral HPLC and by the integration or deconvolution of NMR signals of H_y_-3 recorded in CD_3_OD.

**Figure 2:**
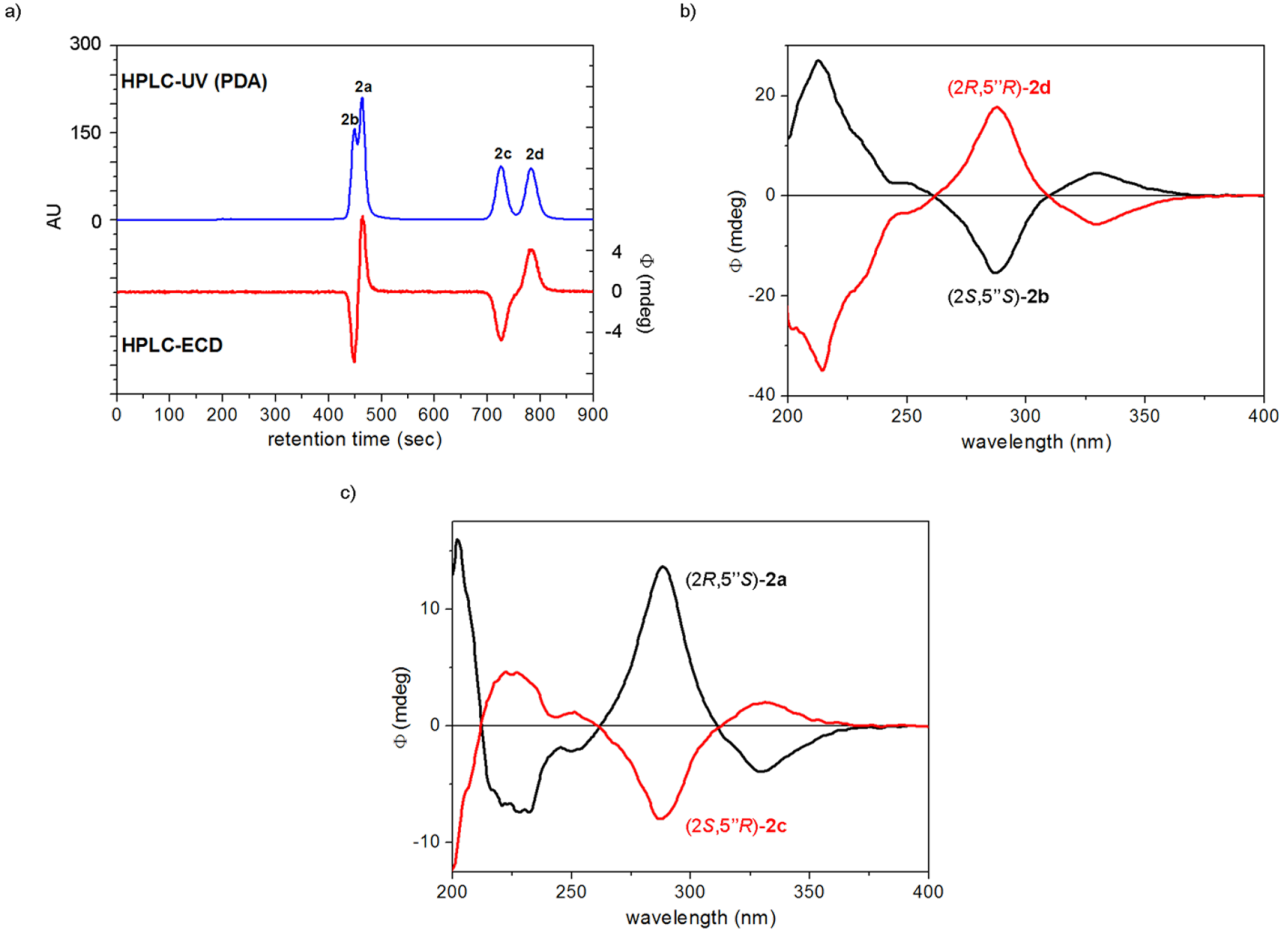
a) Chiral HPLC–UV and HPLC–ECD traces of dracocephins A (**2a**–**d**) using Chiralpak IC column with the eluent MeCN/2-propanol/TFA 97:3:0.1 monitored at 290 nm. b) HPLC–ECD spectra of the first [black: (2*S*,5”*S*)-**2b** or dracocephin A2] and fourth eluted [red: (2*R*,5”*R*)-**2d** or dracocephin A4] stereoisomers of dracocephins A. c) HPLC–ECD spectra of the second [black: (2*R*,5”*S*)-**2a** or dracocephin A1] and third eluted [red: (2*S*,5”*R*)-**2c** or dracocephin A3] stereoisomers of dracocephins A. The absolute configurations were assigned on the basis of the publication of Ren et al. [2].

The separation of the stereoisomers of dracocephins A (±)-**2a**–**d** could be achieved by chiral HPLC using an analytical Chiralpak IC column with the eluent MeCN/2-propanol/TFA 97:3:0.1 (Figure 2).

Four peaks with alternating signs of the ECD signal in the HPLC–UV and –ECD traces were observed, the integration of which showed a near 1:1:1:1 ratio of the stereoisomers. The HPLC–ECD spectra of the separated stereoisomers were recorded, which revealed that the first and fourth eluted stereoisomers are enantiomers, as well as the second and third eluted ones (Figure 2c,d). On the basis of the reported data [2], the (2*S*,5”*S*) and (2*R*,5”*S*) absolute configurations were assigned to the first- and second-eluted stereoisomers **2b** and **2a**, respectively, while the third and fourth eluted **2c** and **2d** stereoisomers had (2*S*,5”*R*) and (2*R*,5”*R*) absolute configuration. The elution order of stereoisomers **2b** and **2a** was inverted in our analysis with Chiralpak IC column compared to the reported separation using Chiralpak-AS-H [2].

In order to confirm the assignment of the absolute configurations for the stereoisomers **2a**–**d** independently from the results of Ren et al. and to test the applicability of the ECD calculations for the stereochemical studies of flavanoid alkaloids, the solution TDDFT–ECD calculation method [6] was applied to **2a**–**d** and **3a**–**d**. For the validation of our computational approach on flavonoids, the ECD spectra of (*R*)-naringenin ((*R*)-**1**), the building blocks of dracocephins A and B, were calculated first with different methods to identify which is able to reproduce most precisely the experimental ECD transitions. ECD and VCD calculations of naringenin have been reported recently by Abbate et al., which could serve as a good basis for our computational studies [7]. Naringenin is a flavanone derivative, whose negative 327 nm n–π* Cotton effect (CE) and the positive 289 nm π–π* CE were correlated with *M* helicity of the fused hetero ring and (2*R*) absolute configuration [8–9]. The mirror image HPLC–ECD spectra of (*R*)- and (*S*)-naringenin were recorded after separating the enantiomers on a Chiralpack IA column with the eluent hexane/2-propanol 80:20 (Supporting Information File 1, Figure S1).

**Figure 3:**
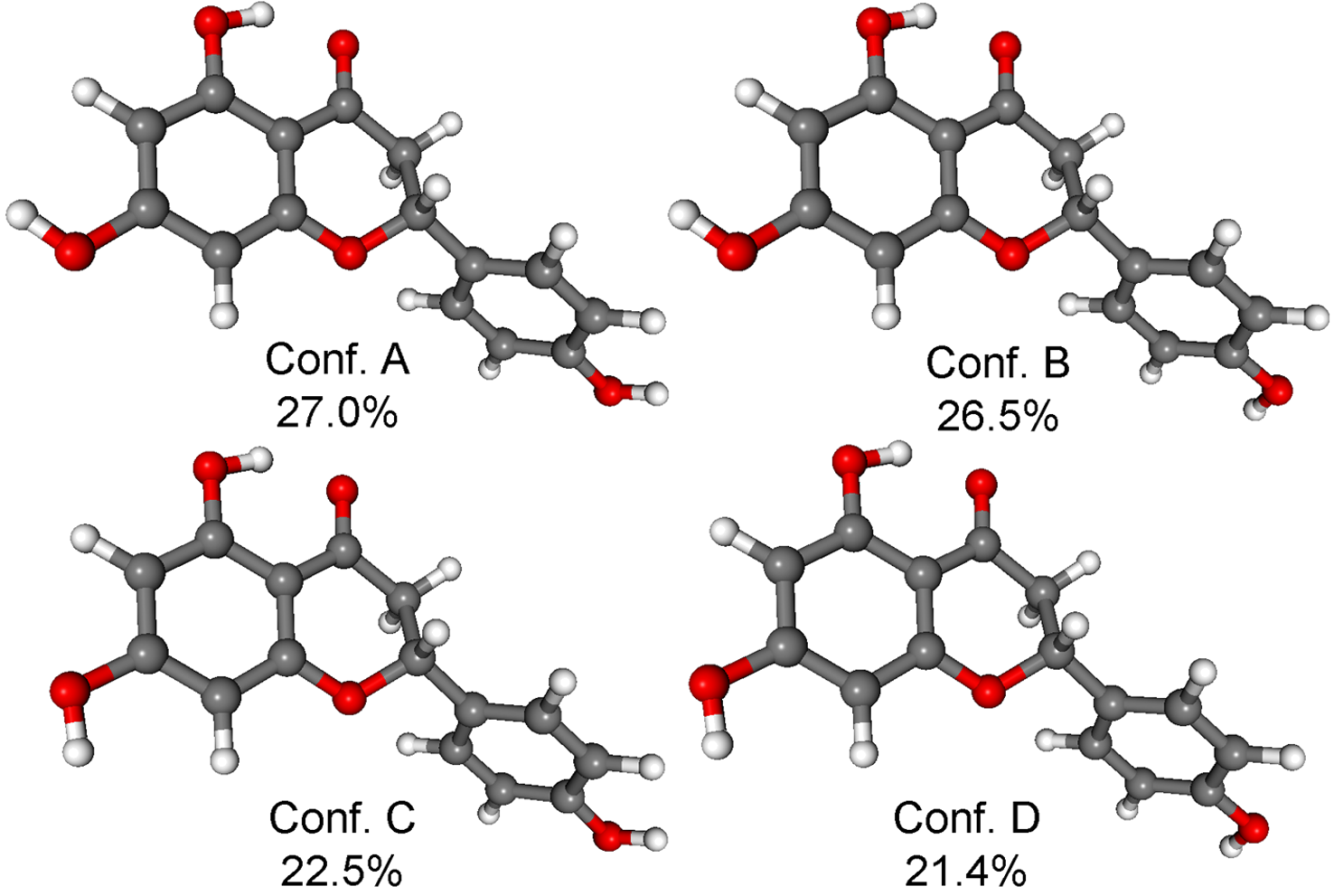
Structure and population of the low-energy CAM-B3LYP/TZVP PCM/CHCl_3_ conformers (>2%) of (*R*)-**1**.

The initial conformational search of (*R*)-**1** with the Merck Molecular Force Field (MMFF) and CHCl_3_ solvent model yielded 8 conformers within a 21 kJ/mol energy window, which were reoptimized at five different DFT levels [B3LYP/6-31G(d) in vacuo, B3LYP/TZVP PCM/CHCl_3_, B97D/TZVP [10–11] PCM/CHCl_3_, CAM-B3LYP/TZVP [12–13] PCM/CHCl_3_ and M06-2X/TZVP [14–15] PCM/CHCl_3_]. While the B3LYP and CAM-B3LYP functionals resulted in 4 low-energy conformers over 2% Boltzmann population with an equatorial C-2 aryl group near coplanar with the C-2– H-2 bond, the B97D and M06-2X ones afforded 8 conformers including four high-energy ones with an axial C-2 aryl group (Supporting Information File 1, Figures S2, S4, S6, and S8). The four CAM-B3LYP/TZVP (PCM/CHCl_3_) conformers (Figure 3) differed mostly in the orientation of the 7- and 4’-OH protons and the condensed hetero ring had *M* helicity with envelope conformation and C-2 pointing out of the plane (ω_C-8a,O-1,C-2,C-3_ = −51.6°, ω_C-8a,C-4a,C-4,C-3_ = 0.4° for the lowest-energy conformer).

The C-2 aryl group was nearly coplanar with the C-2–H-2 bond and the ω_H-2,C-2,C-1’,C-2’_ torsional angle had values in the range of –11.0° to –13.7°. The B97D/TZVP PCM/CHCl_3_ and M06-2X/TZVP reoptimizations produced four high-energy conformers having axial C-2 aryl groups with 34.9% and 32.7% total populations, which apparently overestimated this type of conformers and were not corroborated by the experimental NMR data. Interestingly, the B97D/TZVP PCM method achieved good results with the conformationally flexible 12-membered macrolides, dendrolides A–M [11].

**Figure 4:**
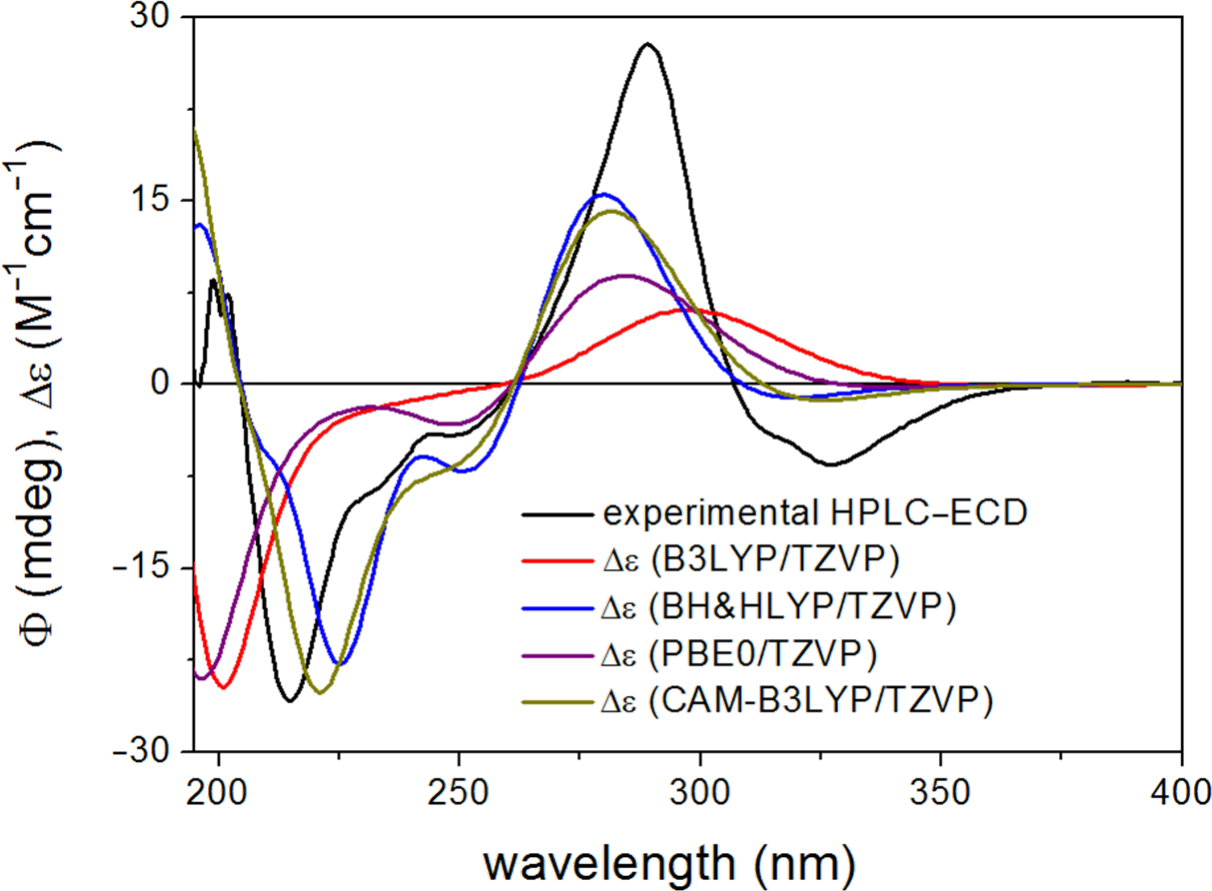
Experimental HPLC–ECD spectrum of (*R*)-naringenin ((*R*)-**1**) compared with the Boltzmann-weighted ECD spectra computed for the CAM-B3LYP/TZVP PCM/CHCl_3_ low-energy conformers of (*R*)-**1** at various levels.

The BH&HLYP, CAM-B3LYP, B3LYP and PBE0 functionals and TZVP basis set were applied for the ECD calculations of the computed conformers, from which only the BH&HLYP and CAM-B3LYP could reproduce the highest-wavelength characteristic n-π* transition, signs, relative intensities and shapes of the other bands, while the other two methods produced much worse agreement (Figure 4).

The results of the ECD calculations of naringenin suggested that the CAM-B3LYP/TZVP (PCM/CHCl_3_) reoptimization of the initial conformers and BH&HLYP/TZVP or CAM-B3LYP/TZVP ECD calculations produce the best agreement and thus these methods were considered primarily in stereochemical ECD studies of **2a**–**d** and **3a**–**d**.

**Figure 5:**
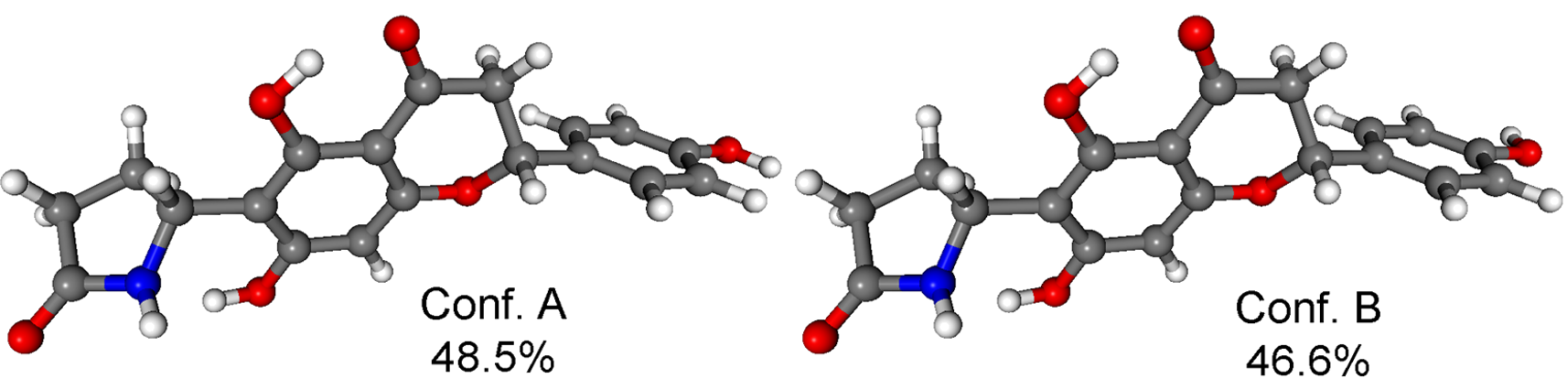
Structure and population of the low-energy CAM-B3LYP/TZVP PCM/MeCN conformers (>2%) of (2*R*,5’’*R*)-**2d**.

**Figure 6:**
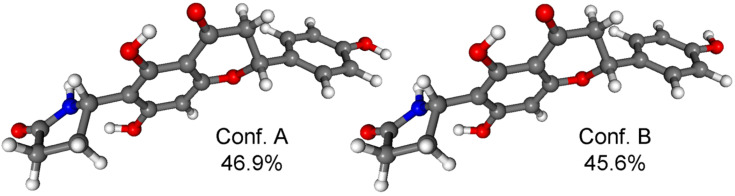
Structure and population of the low-energy CAM-B3LYP/TZVP PCM/MeCN conformers (>2%) of (2*R*,5’’*S*)-**2a**.

The MMFF conformational search of (2*R*,5’’*R*)-**2d** and (2*R*,5’’*S*)-**2a** resulted in 22 and 23 conformers in a 21 kJ/mol energy window, respectively, the reoptimization of which at CAM-B3LYP/TZVP PCM/MeCN level yielded only 2 low-energy conformers over 2% population (Figure 5 and Figure 6).

**Figure 7:**
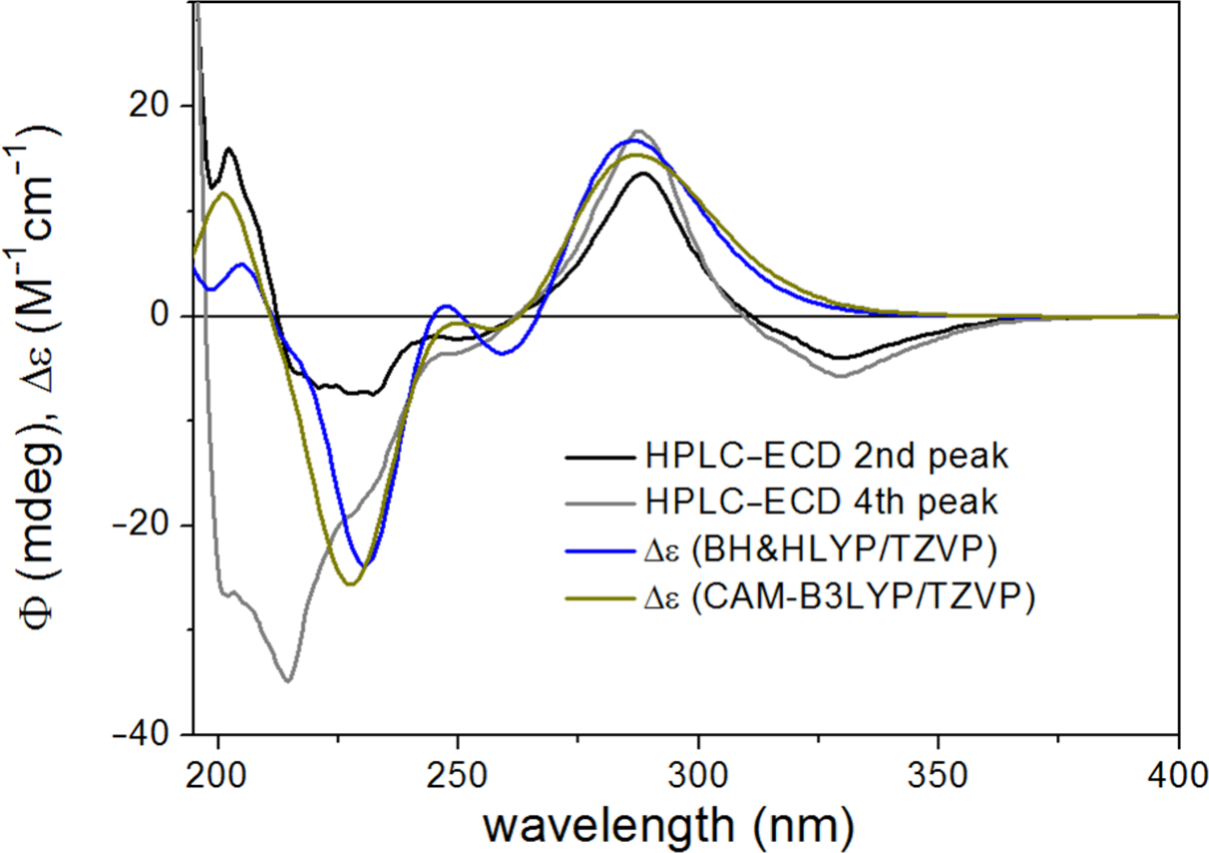
Experimental HPLC-ECD spectra of (2*R*,5’’*S*)-**2a** (second eluted stereoisomer) and (2*R*,5’’*R*)-**2d** (fourth eluted stereoisomer) compared with the Boltzmann-weighted ECD spectra computed for the CAM-B3LYP/TZVP PCM/MeCN low-energy conformers of (2*R*,5’’*R*)-**2** at various levels.

In both stereoisomers, hydrogen bonding between the 7-OH and N–H group fixed the relative orientation of the pyrrolidin-2-one and the naringenin moieties, and this interaction was also responsible for the low number of computed conformers. The two computed conformers had comparable populations and they differed only in the orientation of the 4’-OH. The flavanone moiety adopted a conformation similar to that of naringenin. The Boltzmann-averaged BH&HLYP/TZVP and CAM-B3LYP/TZVP ECD spectra of (2*R*,5’’*R*)-**2d** and (2*R*,5’’*S*)-**2a** confirmed the (2*R*) absolute configuration of the flavanone units, which was also evident from the negative 330 nm n–π* CE. The BH&HLYP/TZVP and CAM-B3LYP/TZVP ECD spectra of (2*R*,5’’*R*)-**2d** showed a better agreement with the ECD of **2a** (second-eluted stereoisomer) on the basis of the agreement with the 202 nm positive CE but the negative 330 nm n–π* transition was missing from the computed ECDs. The negative 330 nm n–π* transition could be only reproduced by the B97D/TZVP conformers (Supporting Information File 1, Figure S15), which, however, contained 28.1% total population of conformers with an axial C-2 aryl group and the overall agreement of their computed ECDs were quite bad. The experimental ECD spectra of the epimers (2*R*,5’’*R*)-**2d** and (2*R*,5’’*S*)-**2a** showed the same negative/positive/negative ECD pattern from 370 nm to 210 nm and they only had a difference in the additional positive CE of (2*R*,5’’*S*)-**2a** at 202 nm (Figure 7).

However, it seemed that (2*R*,5’’*R*)-**2d** had also negative CE below 200 nm, which could not be measured precisely due to the UV cut-off limitation of the 2-propanol eluent. Thus the experimental HPLC–ECD spectra of (2*R*,5’’*R*)-**2d** and (2*R*,5’’*S*)-**2a** only showed differences in the position, shape and relative intensities of the bands, while the patterns of the ECD bands were identical.

**Figure 8:**
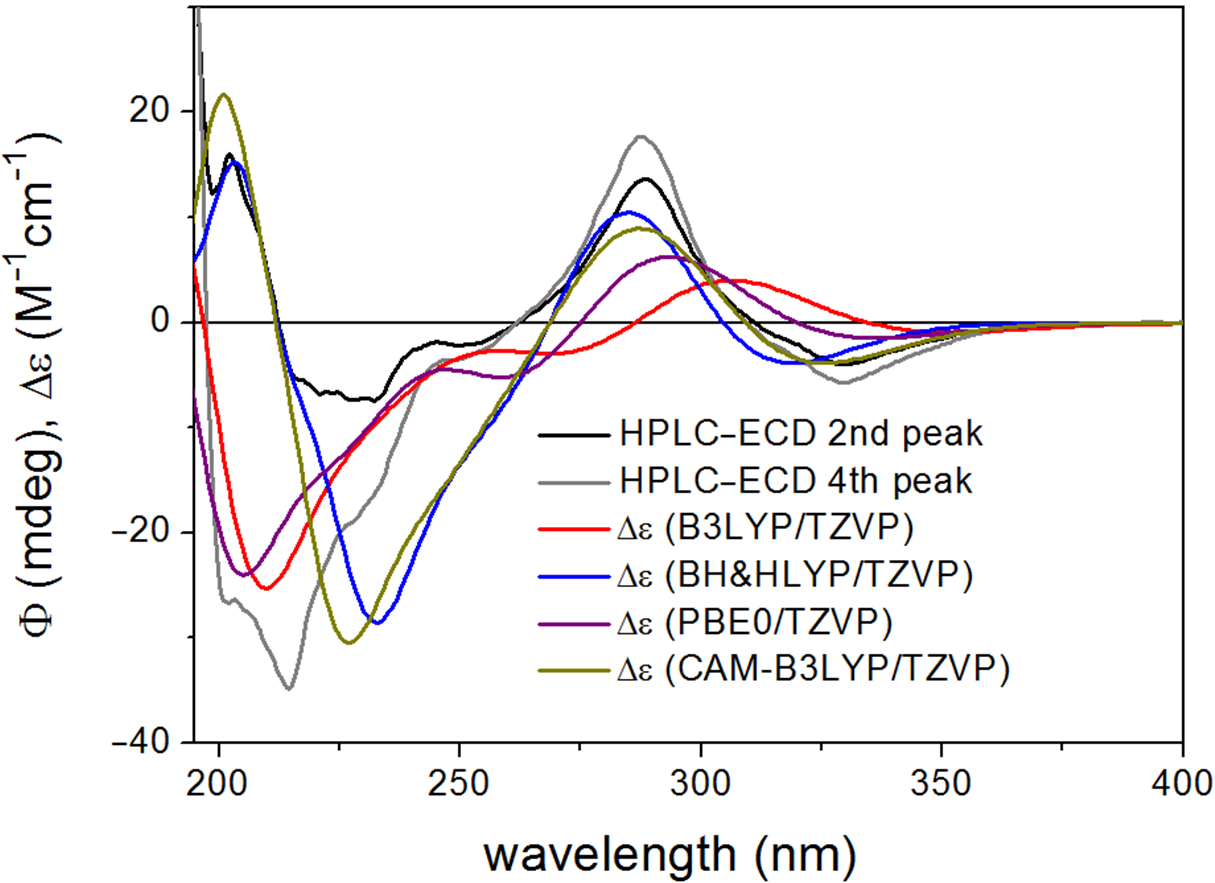
Experimental HPLC–ECD spectra of (2*R*,5’’*S*)-**2a** and (2*R*,5’’*R*)-**2d** compared with the Boltzmann-weighted ECD spectra computed for the CAM-B3LYP/TZVP PCM/MeCN low-energy conformers of (2*R*,5’’*S*)-**2** at various levels.

The Boltzmann-averaged BH&HLYP/TZVP and CAM-B3LYP/TZVP ECD spectra of (2*R*,5’’*S*)-**2a** reproduced well the negative n–π* 330 nm CE and the intense 213 nm negative CE of (2*R*,5’’*R*)-**2d** but it changed the sign of CE to positive below 210 nm, which was characteristic of (2*R*,5’’*S*)-**2a** (Figure 8).

Interestingly, the B3LYP/TZVP and PBE0/TZVP ECD spectra of (2*R*,5’’*S*)-**2a** gave a perfect agreement with the HPLC–ECD of (2*R*,5’’*R*)-**2d** (fourth eluted stereoisomer). All these calculation results would favor the configurational assignment (2*R*,5’’*S*)-**2d** (fourth eluted stereoisomer) and (2*R*,5’’*R*)-**2a** (second eluted stereoisomer), which, however, contradicts the previously published results [2]. From the ECD calculation point of view, the differences in the HPLC ECD spectra of the epimers (2*R*,5’’*R*)-**2d** and (2*R*,5’’*S*)-**2a** were not sufficient and the calculation results were not consistent enough to allow the revision of the previous configurational assignment.

**Figure 9:**
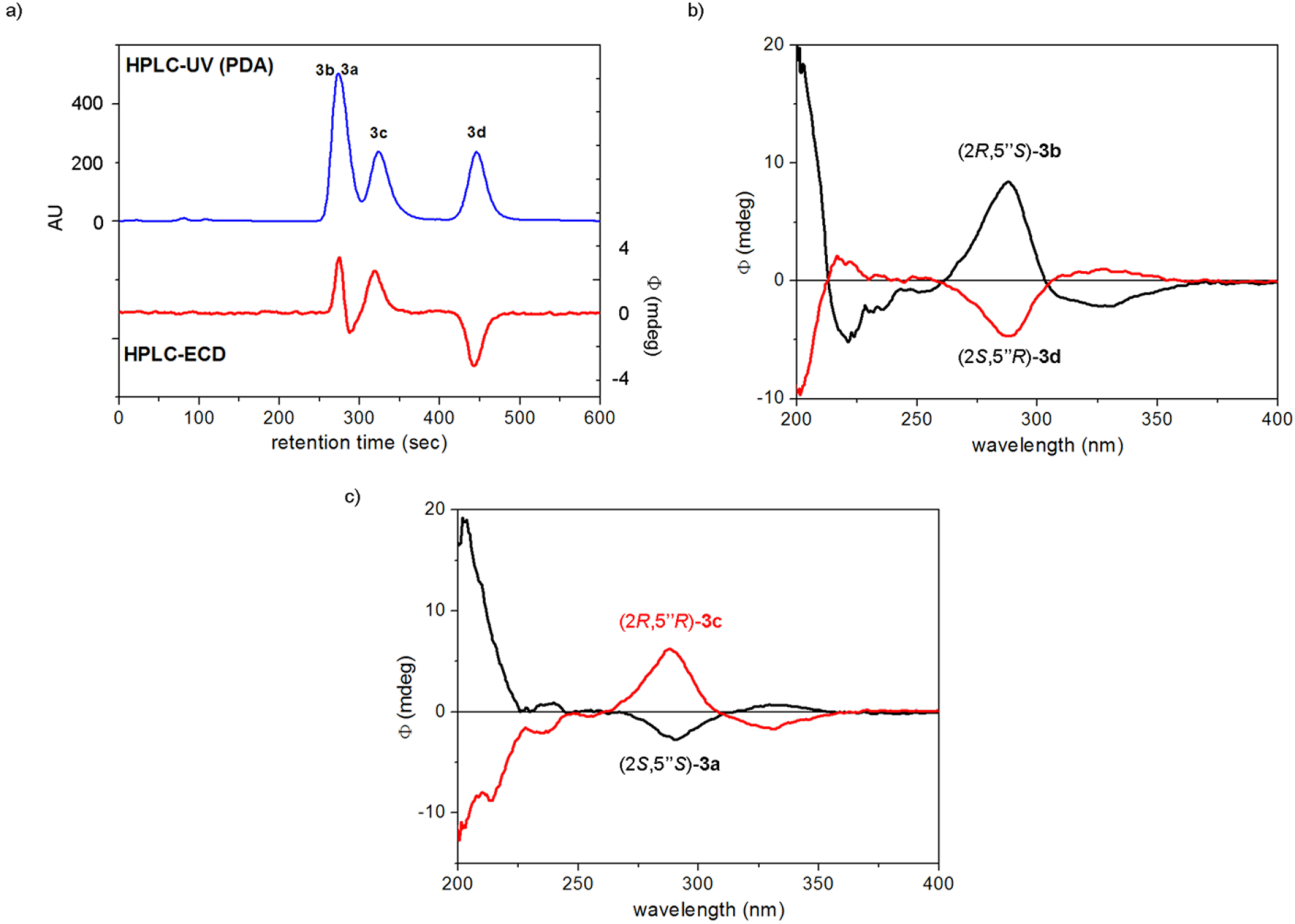
a) Chiral HPLC–UV and HPLC–ECD traces of dracocephins B1–B4 **3a**–**d** using a Chiralpak IC column with the eluent MeCN/2-propanol/TFA 90:10:0.1 monitored at 285 nm. b) HPLC–ECD spectra of the first- [black: (2*R*,5”*S*)-**3b** or dracocephin B2] and fourth eluted [red: (2*S*,5”*R*)-**3d** or dracocephin B4] stereoisomers of dracocephins B. c) HPLC–ECD spectra of the second [black: (2*S*,5”*S*)-**3a** or dracocephin B1] and third eluted [red: (2*R*,5”*R*)-**3c** or dracocephin B3] stereoisomers of dracocephins B. The absolute configurations were assigned on the basis of the publication of Ren et al. [2].

The separation of the stereoisomers of dracocephins B **3a**–**d** could be performed on an analytical Chiralpak IC column using the eluent acetonitrile/2-propanol/trifluoroacetic acid 90:10:0.1 (Figure 9a).

Only partial separation could be achieved for the first two eluting stereosiomers, which was still sufficient to record their HPLC–ECD spectra. The comparison of HPLC–ECD spectra showed that the first and fourth eluted stereoisomers were enantiomers as well as the second and third eluted ones. Similarly to dracochepins A, the elution order was inverted for the first- and second-eluted stereoisomers compared to that of the reported data with Chiralpak AS-H [2]. The solution TDDFT-ECD calculation approach was also applied to the stereoisomers of dracocephins B **3a**–**d** in order to check the applicability of this method.

**Figure 10:**
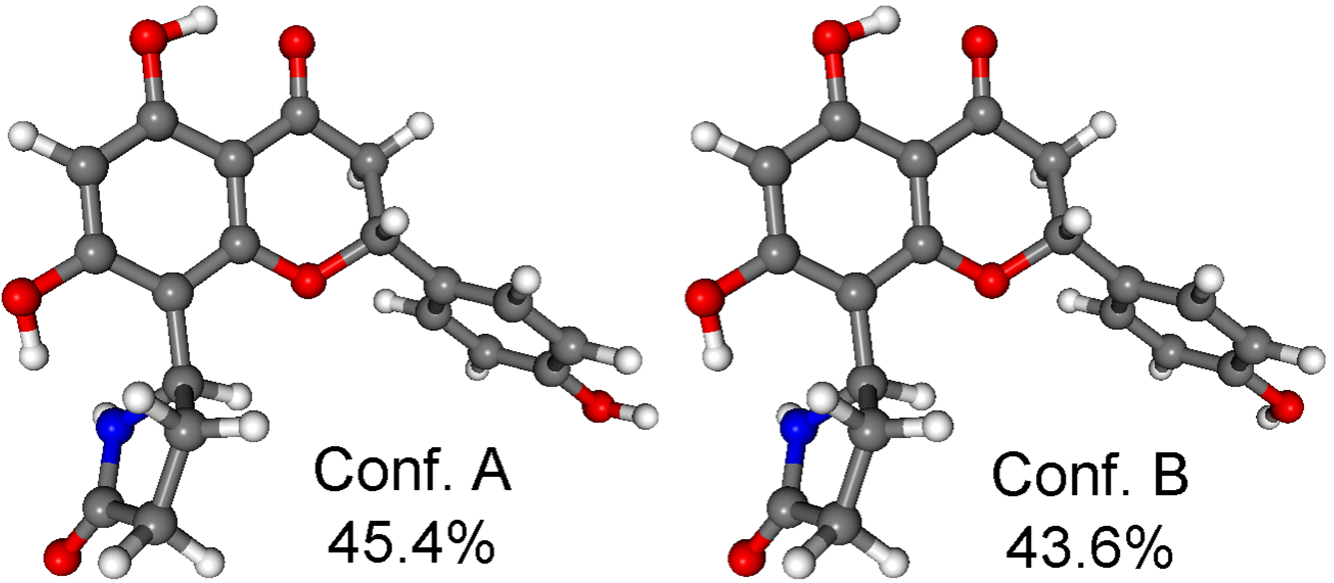
Structure and population of the low-energy CAM-B3LYP/TZVP PCM/MeCN conformers (>2%) of (2*R*,5’’*R*)-**3c**.

**Figure 11:**
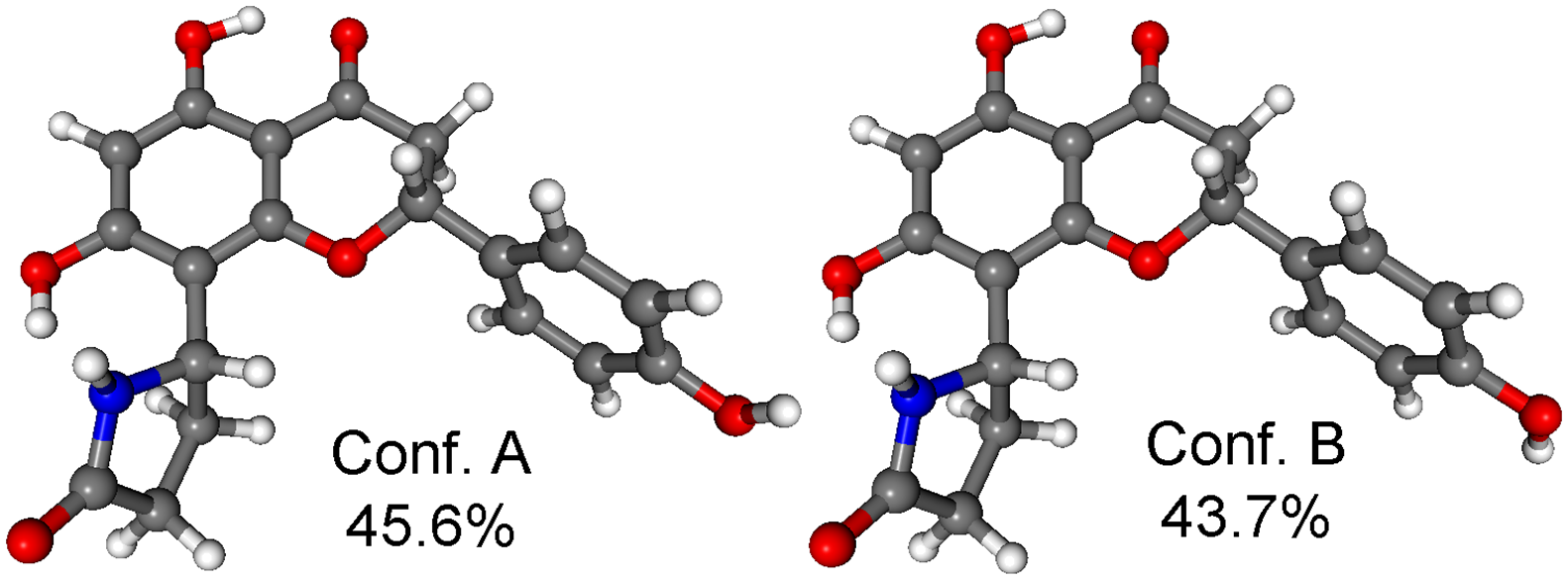
Structure and population of the low-energy CAM-B3LYP/TZVP PCM/MeCN conformers (>2%) of (2*R*,5’’*S*)-**3b**.

The MMFF conformational search of (2*R*,5’’*R*)-**3c** and (2*R*,5’’*S*)-**3b** resulted in 41 and 44 conformers in a 21 kJ/mol energy window, respectively, the reoptimization of which at CAM-B3LYP/TZVP PCM/MeCN level yielded 2 low-energy conformers over 2% population for both stereoisomers (Figure 10 and Figure 11).

Similarly to dracochepins A (**2a**–**d**), hydrogen bonding between the 7-OH and NH groups anchored the orientation of the pyrrolidinone moiety. The two conformers differed in the orientation of the two 4’-OH and the C-2 aryl moiety adopted equatorial orientation with *M* helicity and envelope conformation of the condensed heteroring.

**Figure 12:**
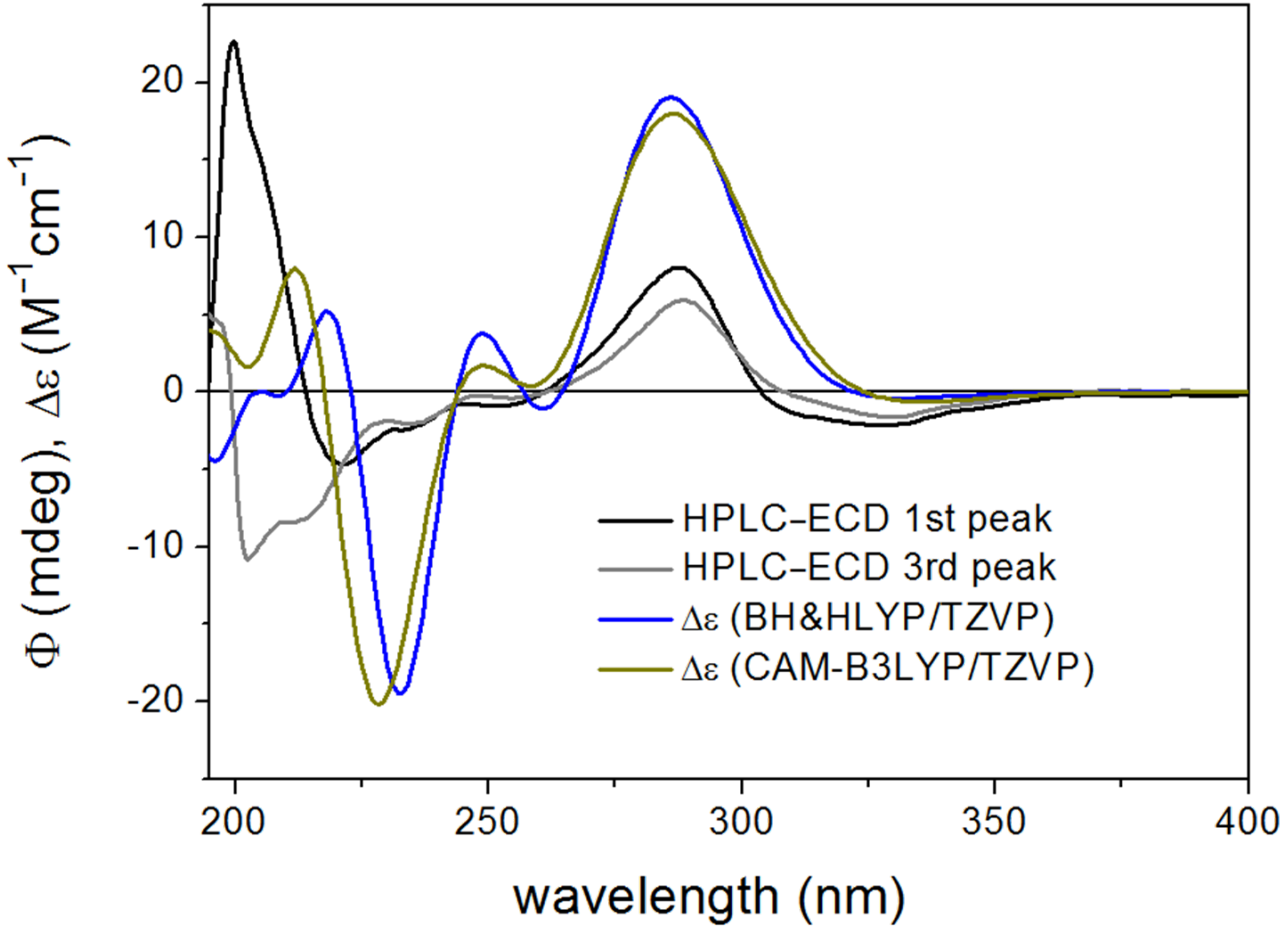
Experimental HPLC-ECD spectra of **3b** (first eluted stereoisomer) and **3c** (third eluted stereoisomer) compared with the Boltzmann-weighted ECD spectra computed for the CAM-B3LYP/TZVP PCM/MeCN low-energy conformers of (2*R*,5’’*R*)-**3** at various levels.

The calculated Boltzmann-averaged ECD spectra of (2*R*,5’’*R*)-**3c** and (2*R*,5’’*S*)-**3b** verified the (2*R*) absolute configuration of the third-eluted and second-eluted stereoisomers on the basis of the high-wavelength negative n–π* and positive π–π* CEs and the BH&HLYP and CAM-B3LYP functionals could reproduce the negative n–π* transition (Figure 12).

**Figure 13:**
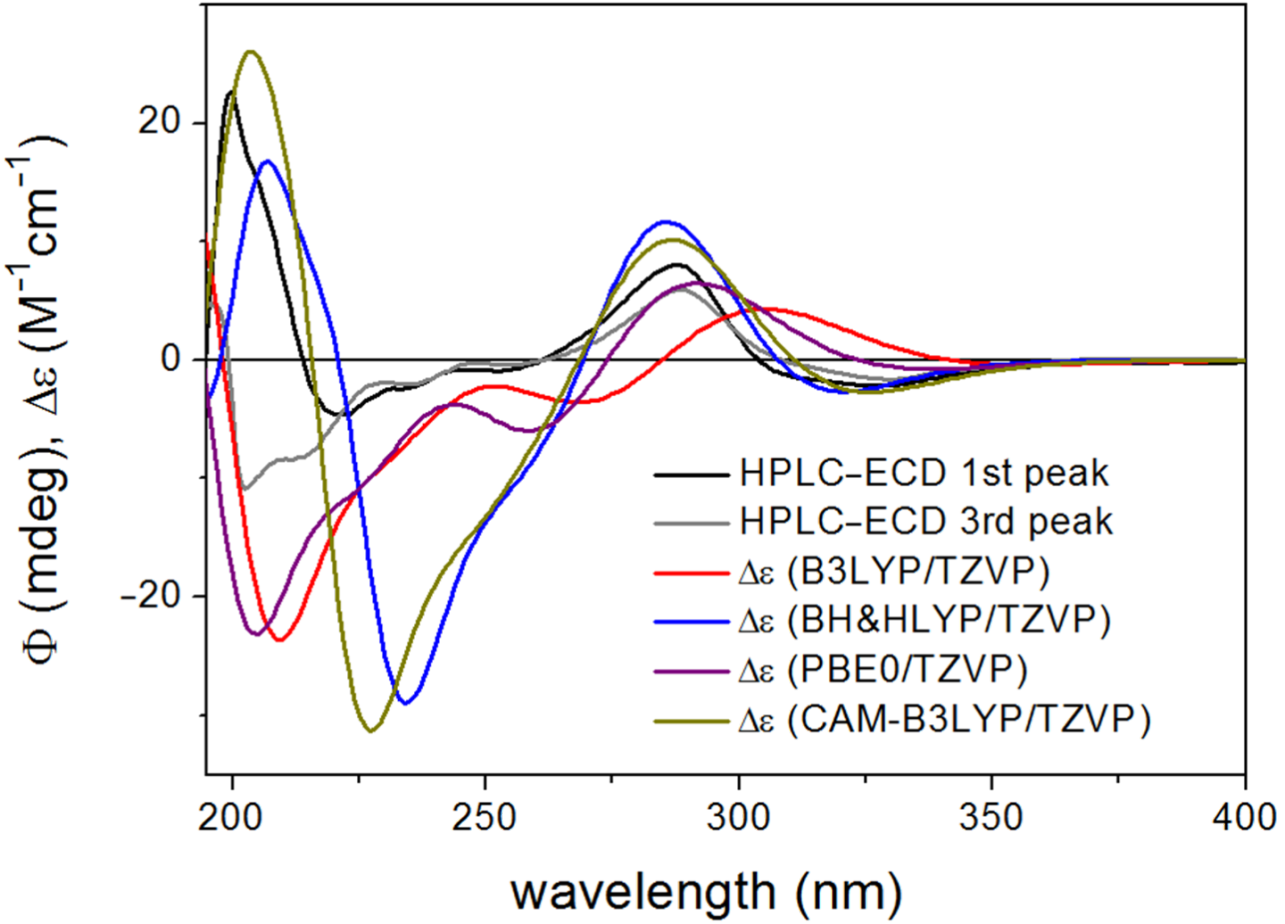
Experimental HPLC-ECD spectra of **3b** and **3c** compared with the Boltzmann-weighted ECD spectra computed for the CAM-B3LYP/TZVP PCM/MeCN low-energy conformers of (2*R*,5’’*S*)-**3** at various levels.

The absolute configuration of C-5” could not be unambiguously deduced from the calculations. The BH&HLYP and CAM-B3LYP ECD spectra of (2*R*,5’’*R*)-**3c** had a weak positive CE at about 250 nm, which was missing from the experimental curve and they rather resembled the HPLC-ECD curve of the first-eluted stereoisomer. The BH&HLYP and CAM-B3LYP ECD spectra of (2*R*,5’’*S*)-**3** reproduced quite well the signs and position of the experimental ECD bands, although the intensity of the 201 negative CE was overestimated (Figure 13).

Similarly to dracocephins A (**2a**–**d**), ECD spectra computed for (2*R*,5’’*S*)-**3** with B3LYP and PBE0 functionals resembled more the experimental HPLC–ECD spectrum of **3c** than that of **3b**.

**Figure 14:**
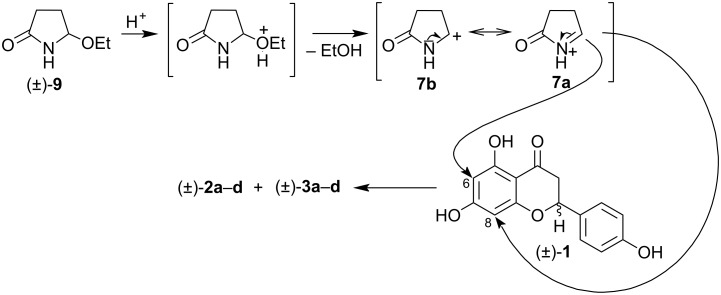
Proposed mechanism for the formation of dracocephins A and B (**2a**–**d** and **3a**–**d**) starting from (±)-**9**.

A plausible mechanism for the formation of dracochepins A and B involves the electrophilic attack of the *N*-acyliminium ion **7a**,**b** on the aromatic ring A of racemic naringenin ((±)-**1**) at C-6 and C-8 (Figure 14).

The electrophilic reagent arises from the amidocarbinol ether (±)-**9** after protonation and loss of ethanol. The planar structure of the cation explains the lack of stereoselectivity observed in the reaction.

### Physicochemical measurements

Physicochemical characterization of dracocephins A and B was also carried out. The relevant calculated (total polar surface area; TPSA) and measured (p*K*_a_, log *P*/*D* and Pe) parameters are shown in Table 1. The lipophilicity of neutral species of naringenin and dracocephins A and B are 3.39, 2.52 and 2.39, respectively. Similarly to this lipophilicity trend, the TPSA value of (±)-naringenin is lower than dracocephins A and B, however, there is no difference between the calculated TPSA values of **2a**–**d** and **3a**–**d**. The p*K*_a_ values of the three phenolic hydroxy groups of the evaluated compounds are quite similar and these are in the range of 6.5 and 12.0. Among these proton-dissociation constants, the lowest acidic p*K*_a_ values are predominant in physiological environment, which is also indicated by the difference of log *D* values between pH 7.4 and pH 6.5. The blood–brain barrier (BBB) specific penetration of dracocephins isomers has also been studied by the PAMPA (parallel artificial membrane permeability assay)-BBB model system, which indicates passive diffusion of test compounds across the brain capillary endothelium. Although naringenin ((±)-**1**) showed a medium penetration characteristic in this model system, dracocephins A (**2a**–**d**) and B (**3a**–**d**) had been described as poorly and practically non-permeable compounds, respectively. These results are in good correlation with a lower lipophilicity (log *P*/*D*_7.4_) and higher TPSA of dracocephins A and B compared to that of naringenin.

**Table 1:** Calculated and measured medicinal chemical parameters of dracocephins A and B compared to those of naringenin.

Compound	p*K*_a_	log *P*	log *D_7.4_*/log *D_6.5_*	TPSA	PAMPA-BBB (P_e_ × 10^–6^ cm/s)

(±)-**1**	6.91 ± 0.06 9.20 ± 0.08 11.85 ± 0.06	3.39 ± 0.01	2.80/3.25	86.99	8.8 ± 0.6 (medium)
**2a**–**d**	6.69 ± 0.01 9.47 ± 0.03 12.34 ± 0.18	2.52 ± 0.02	1.74/2.31	116.1	1.1 ± 0.1 (poorly permeable)
**3a**–**d**	6.54 ± 0.00 9.54 ± 0.00 11.97 ± 0.02	2.39 ± 0.02	1.51/2.12	116.1	non-permeable

### Pharmacological studies

In connection with the BBB penetration studies, **2a**–**d** and **3a**–**d** were tested for their potential towards CNS cytotoxic activity on undifferentiated SH-SY5Y neuroblastoma cells. None of the two isomeric compounds exerted cytotoxicity on this cell line (IC_50_ > 150 μM for both compounds), which rules out their potential CNS antitumor activity and it also suggests them as non-neurotoxic substances. On the other hand, the possibility for neuroprotective activity could not be tested with the applied experimental setup. As an additional bioassay on compounds **2a**–**d** and **3a**–**d**, their potential to interfere with the function of P-glycoprotein (P-gp) was tested. By transporting a wide variety of compounds from the CNS back into the blood stream, P-gp is an important element of the BBB [16]. Naringenin is known to interfere with the BBB penetration of vincristine, a P-gp substrate, through modulating this efflux pump [17], and an additional nitrogen-containing group has the chance to significantly increase P-gp inhibitory activity [18]. Based on the unaltered accumulation of rhodamine 123, a P-gp substrate fluorescent dye, in the P-gp transfected L5178_MDR_ cells, we observed that none of the synthesized compounds inhibits this transporter. The antioxidant activity of dracocephins A and B was also measured, but these compounds, along with naringenin were virtually inactive in the DPPH radical scavenging test (IC_50_ > 200 µg/mL), compared to the positive control quercetin (IC_50_ = 9.4 ± 0.6 µg/mL).

## Conclusion

An efficient one pot synthesis of dracocephins A (**2a**–**d**) and B (**3a**–**d**) was achieved starting from racemic naringenin ((±)-**1**), using the readily accessible *N-*acylaminocarbinol reagent (+)-**9**). The regioisomeric dracochepins A and B could be separated by preparative HPLC and their connectivity was determined on the basis of NMR data. Stereoisomers of dracocephins A (**2a**–**d**) and B (**3a**–**d**) were separated by chiral HPLC and their stereochemistry was studied by TDDFT-ECD calculations. By testing different methods for the calculation of conformers and ECD, the configurational assignment of C-2 of the flavanone moiety could be confirmed, while the C-5” of the pyrrolidinone unit could not be assigned unambiguously. Dracocephins A (**2a**–**d**) and B (**3a**–**d**) were also characterized by physicochemical and in vitro biological measurements. A plausible mechanism of the synthesis of these natural products was also proposed.

## Experimental

### General

Melting points were measured on a SANYO Gallenkamp apparatus and are uncorrected. IR spectra were recorded on a Bruker FTIR instrument. NMR measurements (^1^H, ^13^C, gCOSY, 1D NOESY and 2D NOESY, gHSQCAD, gHMBCAD) were performed on Varian 400 MHz (equipped with 5 mm OneNMR ^15^N-^31^P/{^1^H-^19^F} PFG Probe), Varian 500 MHz (equipped with ^1^H{^13^C/^15^N} 5 mm PFG Triple Resonance ^13^C Enhanced Cold Probe) and Varian 800 MHz (equipped with ^1^H{^13^C/^15^N} Triple Resonance ^13^C Enhanced Salt Tolerant Cold Probe) spectrometers. ^1^H chemical shifts are given on the delta scale as parts per million (ppm) with tetramethylsilane (TMS) as the internal standard (0.00 ppm). ^13^C chemical shifts are given on the delta scale as parts per million (ppm) with tetramethylsilane (TMS) or dimethylsulfoxide-*d*_6_ as the internal standard (0.0 ppm and 39.5 ppm, respectively). MS spectra were recorded on VG-Trio-2 and Finnigan MAT 95SQ instruments using EI or ESI techniques. HRMS analyses were performed on an LTQ FT Ultra (Thermo Fischer Scientific, Bremen, Germany) system. TLC was carried out on TLC Silicagel 60 F_254_ on 20 × 20 aluminium sheets (Merck) and preparative TLC was performed using Silicagel 60 F_254+366_ (Merck) coated glass plates. Column chromatography was performed using Geduran Si 60 (Merck) silica. Racemic naringenin was purchased from Sigma-Aldrich and used without further purification.

### Preparative HPLC

#### Chemicals and reagents

Acetonitrile used in the preparative chromatographic separation was gradient grade LiChrosolv purchased from Merck. The formic acid was reagent grade from Sigma-Aldrich. Water was purified with a Milli-Q system. The sample solvent was spectroscopy grade dimethyl sulfoxide (Uvasol) from Merck.

#### Apparatus

The separation of the isomeric mixtures of flavonoid alkaloids was performed with a Shimadzu chromatograph equipped with an LC-8A pump unit, SPD-M20A Photodiode detector and CBM-20A system controller. The samples were introduced via an SIL-10AP sample injector and the chromatograms were processed using the LCsolution software. The fractions were collected by an FRC10A fraction collector module. The temperature was controlled by a CTO-20AC prominence column oven.

#### Preparative separation procedure

The sample (500 µL/injection) was loaded to a Kinetex 5 µm phenyl-hexyl AXIA packed 21.2 × 150 mm column in the concentration of 20 mg/mL. The separation was achieved with isocratic elution (75% HPLC grade water containing 0.1% formic acid, 25% acetonitrile containing 0.1% formic acid) at 25 °C. The flow rate was 21 mL/min. The fractions were collected based on UV absorption at 220 nm wavelength.

#### Chiral HPLC–ECD analysis

Chiral HPLC separations were carried out with a Jasco HPLC system on Chiralpak IC column (250 × 4.6 mm i.d.; 5 μm) using eluent hexane/acetonitrile 97:3 or 90:10 with 0.1% TFA additive to set the pH to about 2 at a flow rate of 1.0 mL/min for **2** and **3**. HPLC–UV and OR chromatograms were measured with Jasco MD-910 multiwavelength and OR-2090Plus chiral detectors, respectively. The HPLC–ECD traces were recorded at the specified wavelength with a Jasco J-810 CD spectropolarimeter equipped with a 1 cm HPLC flow cell and the baseline was zeroed after the start of each run. The on-line ECD and UV spectra were recorded simultaneously by stopping the flow at the UV absorption maximum of each peak. ECD ellipticity values (Φ) were not corrected for concentration. For an HPLC–ECD spectrum, three consecutive scans were recorded and averaged with 2 nm bandwidth, 1 s response, and standard sensitivity. The HPLC–ECD spectrum of the eluent was recorded in the same way. The concentration of the injected sample was set so that the HT (voltage) value did not exceed 500 V in the HT channel.

**Dracocephins A1–A4 (2ª**–**d**)**: 2b** (first eluted stereoisomer): *t*_R_ = 7.26 min, λ [nm] (Φ)}: 330 (4.43), 288 (–15.34), 249sh (2.48), 230 (11.60), 213 (27.01). **2a** (second eluted stereoisomer): *t*_R_ = 7.49 min, λ [nm] (Φ)}: 329 (–3.95), 289 (13.60), 250sh (–2.18), 227 (–7.35), 202 (15.98). **2c** (third eluted stereoisomer): *t*_R_ = 11.49 min, λ [nm] (Φ)}: 329 (1.92), 289 (–7.86), 250sh (1.09), 227 (4.56), 202 (–10.49). **2d** (fourth eluted stereoisomer): *t*_R_ = 12.38 min, λ [nm] (Φ)}: 330 (–5.71), 288 (17.65), 249sh (–3.54), 230sh (–17.68), 213 (–33.89).

**Dracocephins B1–B4** (**3a–d**)**: 3b** (first peak): *t*_R_ = 4.87 min, λ [nm] (Φ)}: 327 (–2.15), 288 (8.40), 252sh (–0.88), 234sh (–2.24), 221 (–5.07), 201 (19.79). **3a** (second peak): *t*_R_ = 4.87 min, λ [nm] (Φ)}: 332 (0.65), 290 (–2.74), 254 (0.13), 240sh (0.90), 203 (18.81). **3c** (third peak): *t*_R_ = 5.53 min, λ [nm] (Φ)}: 328 (–1.02), 288 (6.25), 254 (–0.42), 235sh (–2.14), 203 (–11.47). **3d** (fourth peak): *t*_R_ = 7.56 min, λ [nm] (Φ)}: 328 (1.02), 288 (–4.68), 252sh (0.17), 234sh (0.42), 221 (1.46), 201 (–9.10).

**(±)-Naringenin ((±)-1):** (2*R*)*-***1** (first peak) *t*_R_ = 5.19 min on a Chiralpak IA column (hexane/2-propanol 80:20), λ [nm] (Φ)}: 327 (–6.75), 314sh (–4,02), 289 (28.91), 235sh (–7.96), 215 (–28.48); (2*S*)-**1** (second peak): *t*_R_ = 5.84 min on a Chiralpak IA column (hexane/2-propanol 80, λ [nm] (Φ)}: 327 (5.49), 314sh (3.49), 289 (–22.65), 235sh (4.69), 215 (21.67).

### Preparation of dracocephins A ((±)-**2a–d**) and B ((±)-**3a–d**)

To a suspension of racemic naringenin [(±)-**1**] (200 mg, 0.735 mmol) in nitromethane (5 mL) was added 5-ethoxypyrrolidine-2-one ((±)-**9**, 114 mg, 0.883 mmol), a catalytic amount of *p*-toluenesulfonic acid and the mixture was refluxed for 4 h. The solvent was evaporated in vacuo and the solid residue was purified by column chromatography (CH_2_Cl_2_/CH_3_OH = 10:1). An isomeric mixture of (±)-**2a**–**d** and (±)-**3a**–**d** (168 mg, 64%) was obtained as a white crystalline solid, *R*_f_ (CH_2_Cl_2_:CH_3_OH = 10:1) 0.32. A sample of the mixture (47 mg) was subjected to further purification by preparative HPLC, which gave title compounds (±)-**2a**–**d** (16 mg, 99.66% purity) as a white solid, mp 168–170 °C; IR (KBr) *ν*_max_: 3393, 3034, 2970, 1634, 1519, 1455, 1340, 1310, 1279, 1170, 1090 cm^−1^; ^1^H NMR (799.7 MHz, CD_3_OD) δ_H_ 2.21–2.27 (m, 1H, H_x_-4”), 2.36–2.42 (m, 1H, H_x_-3”), 2.43–2.49 (m, 1H, H_y_-4”), 2.59–2.64 (m, 1H, H_y_-3”), 2.71–2.75 (m, 1H, H_x_-3), 3.12 and 3.13 [sum 1H, (dd, *J* = 17.0, 12.8 Hz, H_y_-3_A_) and (dd, *J* = 17.0, 12.6 Hz, H_y_-3_B_), respectively], 5.33–5.36 (buried m, 2H, H-5” and H-2), 5.95 (s, 1H, H-8), 6.80–6.82 (m, 2H, H-3’ and H-5’), 7.29–7.32 (m, 2H, H-2’ and H-6’); ^13^C NMR (201.1 MHz, CD_3_OD) δ_C_ 26.7 (C-4”), 32.07 and 32.10 (C-3”), 43.96 and 43.99 (C-3), 49.4 (C-5”), 80.52 and 80.53 (C-2), 96.04 and 96.06 (C-8), 103.1 (C-10), 109.44 and 109.46 (C-6), 116.4 (C-3’ and C-5’), 129.09 and 129.10 (C-2’ and C-6’), 131.0 (C-1’), 159.12 and 159.13 (C-4’), 163.47 and 163.50 (C-5), 163.90 and 163.91 (C-9), 166.8 (C-7), 181.60 and 181.63 (C-2”), 198.1 (C-4); HRMS [M + H] calcd for C_19_H_18_O_6_N: 356.11286; found: 356.11284; ESI–MS–MS (CID = 35%) (rel. int. %): 339(100); 338(4); 311(2); 236(3); and title compounds (±)-**3a**–**d** (21 mg, 99.92% purity), also a white solid, mp 238–240 °C; IR (KBr) *ν*_max_: 3416, 3034, 2970, 1630, 1519, 1447, 1378, 1343, 1257, 1176, 1081 cm^−1^; ^1^H NMR (799.7 MHz, CDCl_3_:CD_3_OD = 2:1) δ_H_ 2.15–2.29 (buried m, 2H, H_x_-3” and H_x_-4”), 2.30–2.37 (m, 1H, H_y_-3”), 2.38–2.46 (m, 1H, H_y_-4”), 2.73–2.79 (m, 1H, H_x_-3), 3.09 and 3.14 [sum 1H, (dd, *J* = 17.0, 13.1 Hz, H_y_-3_A_) and (dd, *J* = 17.1, 13.2 Hz, H_y_-3_B_), respectively], 5.28–5.37 (buried m, 2H, H-5” and H-2), 6.00 (s, 1H, H-6), 6.87–6.90 (m, 2H, H-3’ and H-5’), 7.28–7.31 (m, 2H, H-2’ and H-6’); ^13^C NMR (201.1 MHz, CD_3_OD) δ_C_ 25.8 and 25.9 (C-4”), 31.1 (C-3”), 42.8 and 43.5 (C-3), 48.4 and 48.6 (C-5”), 79.7 and 80.0 (C-2), 96.6 (C-6), 102.6 (C-10), 107.9 (C-8), 115.9 (C-3’ and C-5’), 128.2 (C-2’ and C-6’), 129.3 (C-1’), 158.0 (C-4’), 161.9 (C-9), 163.4* (C-5), 165.6* (C-7), 180.2 (C-2”), 196.9 and 197.0 (C-4); (*: may be reversed); HRMS [M + H]: calcd for C_19_H_18_O_6_N 356.11286; found: 356.11285; ESI–MS–MS (CID = 35%) (rel. int. %): 339 (100); 338 (4); 311 (3); 236 (6).

## Supporting Information

File 1 Details of physicochemical measurements, biology and computational section, computed ECD spectra, conformers of compounds **1**–**3** and NMR spectra of compounds **2** and **3**.
