# Supplementary material for: Biomimetic synthesis and HPLC–ECD analysis of the isomers of dracocephins A and B
Source: Beilstein J Org Chem. 2016 Nov 24;12:2523–34. doi: 10.3762/bjoc.12.247 (PMC5238559; doi:10.3762/bjoc.12.247)
Supplement: File 1 — Details of physicochemical measurements, biology and computational section, computed ECD spectra, conformers of compounds 1–3 and NMR spectra of compounds 2 and 3. [file Beilstein_J_Org_Chem-12-2523-s001.pdf]

Supporting Information

for

Biomimetic synthesis and HPLC–ECD analysis of the isomers of dracocephins A and B

Viktor Ilkei,[1] András Spaits,[1] Anita Prechl,[2] Áron Szigetvári,[2] Zoltán Béni,[2] Miklós Dékány,[2] Csaba Szántay Jr.,[2] Judit Müller,[2] Árpád Könczöl,[2] Ádám Szappanos,[3] Attila Mándi,*[3] Sándor Antus,[3] Ana Martins,[4,5] Attila Hunyadi,[6] György Tibor Balogh,[2] György Kalaus (†),[1] Hedvig Bölcskei,[1] László Hazai*[1] and Tibor Kurtán*[3]

Details of physicochemical measurements, biology and computational section, computed ECD spectra, conformers of compounds 1–3 and NMR spectra of compounds 2 and 3.

Table of contents

Details of physicochemical measurements

Biology

Computational section

Figure S1: HPLC–ECD spectra of (2*R*)- and (2*S*)-naringenin (**1**).

Figure S2: Structure and population of the low-energy B3LYP/6-31G(d) in vacuo conformers (>2%) of (*R*)-**1**.

Figure S3: Experimental HPLC–ECD spectrum of (*R*)-**1** compared with the Boltzmann-weighted ECD spectra computed for the B3LYP/6-31G(d) in vacuo low-energy conformers at various levels.

Figure S4: Structure and population of the low-energy B3LYP/TZVP PCM/$CHCl_3$ conformers (>2%) of (*R*)-**1**.

Figure S5: Experimental HPLC–ECD spectrum of (R)-1 compared with the Boltzmann-weighted ECD spectra computed for the B3LYP/TZVP PCM/CHCl3 low-energy conformers at various levels.

Figure S6: Structure and population of the low-energy B97D/TZVP PCM/$CHCl_3$ conformers (>2%) of (*R*)-**1**.

Figure S7: Experimental HPLC–ECD spectrum of (*R*)-**1** compared with the Boltzmann-weighted ECD spectra computed for the B97D/TZVP PCM/$CHCl_3$ low-energy conformers at various levels.

Figure S8: Structure and population of the low-energy M06-2X/TZVP PCM/$CHCl_3$ conformers (>2%) of (*R*)-**1**.

Figure S9: Experimental HPLC–ECD spectrum of (*R*)-**1** compared with the Boltzmann-weighted ECD spectra computed for the M06-2X/TZVP PCM/$CHCl_3$ low-energy conformers at various levels.

Figure S10: Experimental HPLC–ECD spectra of **2a** and **2d** compared with the Boltzmann-weighted ECD spectra computed for the CAM-B3LYP/TZVP PCM/MeCN low-energy conformers of (2*R*,5''*R*)-**2** at various levels.

Figure S11: Experimental HPLC–ECD spectra of **2a** and **2d** compared with the Boltzmann-weighted ECD spectra computed for the B3LYP/6-31G(d) in vacuo low-energy conformers of (2*R*,5''*R*)-**2** at various levels.

Figure S12: Experimental HPLC–ECD spectra of **2a** and **2d** compared with the Boltzmann-weighted ECD spectra computed for the B3LYP/6-31G(d) in vacuo low-energy conformers of (2*R*,5''*S*)-**2** at various levels.

Figure S13: Experimental HPLC–ECD spectra of **2a** and **2d** compared with the Boltzmann-weighted ECD spectra computed for the B3LYP/TZVP PCM/MeCN low-energy conformers of (2*R*,5''*R*)-**2** at various levels.

Figure S14: Experimental HPLC–ECD spectra of **2a** and **2d** compared with the Boltzmann-weighted ECD spectra computed for the B3LYP/TZVP PCM/MeCN low-energy conformers of (2*R*,5''*S*)-**2** at various levels.

Figure S15: Experimental HPLC–ECD spectra of **2a** and **2d** compared with the Boltzmann-weighted ECD spectra computed for the B97D/TZVP PCM/MeCN low-energy conformers of (2*R*,5''*R*)-**2** at various levels.

Figure S16: Experimental HPLC–ECD spectra of **2a** and **2d** compared with the Boltzmann-weighted ECD spectra computed for the B97D/TZVP PCM/MeCN low-energy conformers of (2*R*,5''*S*)-**2** at various levels.

Figure S17: Experimental HPLC–ECD spectra of **3b** and **3c** compared with the Boltzmann-weighted ECD spectra computed for the CAM-B3LYP/TZVP PCM/MeCN low-energy conformers of (2*R*,5''*R*)-**3** at various levels.

Figure S18: Experimental HPLC–ECD spectra of **3b** and **3c** compared with the Boltzmann-weighted ECD spectra computed for the B3LYP/6-31G(d) in vacuo low-energy conformers of (2*R*,5''*R*)-**3** at various levels.

Figure S19: Experimental HPLC–ECD spectra of **3b** and **3c** compared with the Boltzmann-weighted ECD spectra computed for the B3LYP/6-31G(d) in vacuo low-energy conformers of (2*R*,5''*S*)-**3** at various levels.

Figure S20: Experimental HPLC–ECD spectra of **3b** and **3c** compared with the Boltzmann-weighted ECD spectra computed for the B3LYP/TZVP PCM/MeCN low-energy conformers of (2*R*,5''*R*)-**3** at various levels.

Figure S21: Experimental HPLC–ECD spectra of **3b** and **3c** compared with the Boltzmann-weighted ECD spectra computed for the B3LYP/TZVP PCM/MeCN low-energy conformers of (2*R*,5''*S*)-**3** at various levels.

Figure S22: ^{1}H NMR spectrum of **(±)-3a–d** in CD_3OD

Figure S23: NOESY spectrum of **(±)-3a–d** (No. 1)

Figure S24: NOESY spectrum of **(±)-3a–d** (No. 2)

Figure S25: gCOSY spectrum of **(±)-3a–d**

Figure S26: ^{1}H NMR spectrum of **(±)-3a–d** in $CDCl_3$: CD_3OD (2:1)

Figure S27: ^{1}H NMR spectrum of **(±)-3a–d** in $CDCl_3$: CD_3OD (2:1) – zoom from 5.24 to 7.74 ppm

Figure S28: ^{1}H NMR spectrum of **(±)-3a–d** in $CDCl_3$: CD_3OD (2:1) – zoom from 2.04 to 4.20 ppm

Figure S29: gHSQCad spectrum of **(±)-3a–d**

Figure S30: gHMBCad spectrum of **(±)-3a–d**

Figure S31: ^{1}H NMR spectrum of **(±)-2a–d**

Figure S32: ^{1}H NMR spectrum of **(±)-2a–d** – zoom from 5.30 to 7.38 ppm

Figure S33: ^{1}H NMR spectrum of **(±)-2a–d** – zoom from 2.14 to 3.50 ppm

Figure S34: gCOSY spectrum of **(±)-2a–d**

Figure S35: NOESY spectrum of **(±)-2a–d**

Figure S36: gHSQCad spectrum of **(±)-2a–d**

Figure S37: gHMBCad spectrum of **(±)-2a–d**

Figure S38: ^{13}C NMR spectrum of **(±)-2a–d**

Details of physicochemical measurements

Potentiometric pK_a determination

T3-Sirius automated pK_a analyser (Sirius Analytical Instruments Ltd., Forest Row, UK) fitted with combina,tion Ag/AgCl pH electrode was used for determination of dissociation constants. The pK_a values were calculated by RefinementProTM software (Sirius Analytical Instruments Ltd., Forest Row, UK). Methodologies used by the software have been described in earlier publications [1,2].

Titration of dracocephins A and B

The titration of dracocephins A and B was carried out in a similar procedure as described in the literature [3]. In each experiment, 1.50 mL of a 1 mM aqueous solution of sample was prealkalified to pH 12.0–12.5 with 0.5 M KOH, and then titrated with 0.5 M HCl to an appropriately low pH, usually 2.0. The titrations were carried out at constant ionic strength (I = 0.15 M KCl) and temperature (T = 25.0 ± 0.1 °C), and under nitrogen atmosphere. Three to five parallel measurements were carried out and the pK_a values of samples were calculated by RefinementProTM software.

Potentiometric determination of partition coefficients

Partition coefficients were determined in a similar manner as described in the literature [4]. Typically, 0.8–2.5 mL of 0.5–10 mM solutions of samples were titrated under the same conditions as in pK_a determinations but in the presence of various amounts of the partitioning solvent, water-saturated *n*-octanol. The phase ratio applied was varied from 1.5 mL water – 0.05 mL *n*-octanol to 1.5 mL water – 1.0 mL

n-octanol. From the *n*-octanol containing titrations the p_oK_a (the apparent ionization constant in the presence of *n*-octanol) and then log*P* values were estimated and refined by a weighted non-linear least-squares procedure, where the aqueous pK_a values (taken from aqueous titrations) were used as unrefined contributions. For each compound a minimum of three to six titrations at different phase volume ratios were measured, and the respective average log*P* values were calculated. log*D* values were also calculated by log*P* and pK_a values using the Henderson–Hasselbalch equation. The relevant relationships between log*P*, pK_a, and p_oK_a, for mono- and multiprotic substances, including cases of ion-pair formation, have been described in detail earlier [5].

Antioxidant activity: The radical scavenging capacity of dracocephins A and B were tested in the microplate format of the 2,2-diphenyl-1-picrylhydrazyl (DPPH) assay [6]. Naringenin and quercetin were obtained from PhytoLab (Vestenbergsgreuth, Germany), and used as controls.

Permeability measurements: The PAMPA-BBB method was previously published [7]. Briefly, a 96-well acceptor plate and a 96-well filter plate are assembled into a sandwich. The hydrophobic filter material of the 96 well filter plate is coated with 5 µL of a 2.6% (w/v) dodecane/hexane (25:75 v/v %) solution of porcine brain lipid (PBL). Subsequently, the acceptor wells at the bottom of the sandwich are filled with 300 µL of 10 mM PBS solution with 5% DMSO adjusted to pH 7.4. The donor wells at the top of the sandwich are hydrated with 150 µL of test compound solution. The test compound solution is prepared by diluting ×100 from a 10 mM stock solution in DMSO using PBS solution at pH 7.4 with 5% DMSO followed by filtration through a MultiScreen Solubility filter plate. The resulting sandwich is then incubated at 37 °C

for 4 h. After the incubation, PAMPA sandwich plates are separated and compound concentrations in donor and acceptor solutions are determined by HPLC-DAD.

Biology

Cell lines

SH-SY5Y neuroblastoma was cultured in EMEM media supplemented with non-essential amino acids, 1 mM Na pyruvate and 10% inactivated fetal bovine serum, nystatin, 2 mM L-glutamine, 100 U penicillin and 0.1 mg streptomycin, purchased from Sigma. Cells were cultured at 37 °C and 5% CO_2.

Two mouse lymphoma cell lines were used to test for the capacity of the compounds to inhibit the function of the ABCB1 transporter: a parental ($L5178_{PAR}$) cell line, L5178 mouse T-cell lymphoma cells (ECACC catalog no. 87111908, U.S. FDA, Silver Spring, MD, U.S.), and a multi-drug resistant ($L5178_{MDR}$) cell line derived from L5178 by transfection with pHa MDR1/A retrovirus [8]. Cells were cultured in McCoy's 5A media supplemented inactivated horse serum and antibiotics as above. MDR cell line was selected by culturing the infected cells with 60 μg/L colchicine (Sigma).

Materials and methods:

Compounds (±)-**2a**–**d** and (±)-**3a**–**d** were dissolved in DMSO at a stock concentration of 10 mM.

Cytotoxicity on SH-SY5Y cells: Cytotoxicity on SH-SY5Y cells was measured in an analogous manner as described in the literature [9]. 10,000 cells per well were seeded overnight. Serial dilutions of the compounds were prepared and added the following day to the plate. Cells were then incubated for 48 h, after which 10% MTT was added to each well. After 4 h, SDS was added to the medium and the results

were read after o/n incubation. Fifty percent inhibitory concentrations (IC_{50}) were calculated using nonlinear regression curve fitting of log (inhibitor) versus normalized response and variable slope with a least squares (ordinary) fit of GraphPad Prism 5 software, for three independent samples.

Evaluation of compounds effect on rhodamine 123 accumulation: Rd123 concentration inside $L5178_{PAR}$ and $L5178_{MDR}$ cells was determined by flow cytometry. Briefly, 2×10^6 cells/mL were treated with 2 and 20 μM of each compound and incubated for 10 min at rt. Rhodamine 123 (Sigma, Germany) was added to a final concentration of 5.2 μM. The samples were incubated for 20 min at 37 °C in water bath and then centrifuged (2000 rpm, 2 min). The pellet was resuspended in 0.5 mL of phosphate buffer saline (PBS) (Sigma, Germany). The washing step was repeated twice. The fluorescence of the samples was measured by flow cytometry (Becton Dickinson FACScan, BD, U.S.). Tariquidar at 2 μM was used as positive control. Fluorescence activity ratio measures the capacity of inhibition (accumulation of Rd123) and it is equal to the ratio between the FL-1 values of the $L5178_{MDR}$ cells treated and untreated.

Computational section

Mixed torsional/low-frequency mode conformational searches were carried out by means of the Macromodel 9.9.223 software using the Merck Molecular Force Field (MMFF) with an implicit solvent model for $CHCl_3$ [10]. Geometry reoptimizations were carried out at the B3LYP/6-31G(d) level in vacuo, B3LYP/TZVP, B97D/TZVP, CAM-B3LYP/TZVP and M06-2X/TZVP levels with the PCM solvent model for MeCN or $CHCl_3$. TDDFT ECD calculations were run with various functionals (B3LYP, BH&HLYP, CAM-B3LYP, PBE0) and the TZVP basis set as implemented in the Gaussian 09 package with the same or no solvent model as in the preceding DFT

optimization step [11]. ECD spectra were generated as sums of Gaussians with 3000 cm^{-1} widths at half-height (corresponding to ca. 24 nm at 280 nm), using dipole-velocity-computed rotational strength values [12]. Boltzmann distributions were estimated from the ZPVE-corrected B3LYP/6-31G(d) energies in the gas-phase calculations and from the B3LYP/TZVP, B97D/TZVP, CAM-B3LYP/TZVP and M06-2X/TZVP energies in the solvated ones. The MOLEKEL software package was used for visualization of the results [13].

References

1. Avdeef, A. *J. Pharm. Sci.* **1993**, *82*, 183–190.
2. Allen, R. I.; Box, K. J.; Comer, J. E. A.; Peake, C.; Tam, K. Y. *J. Pharm. Biomed. Anal.* **1998**, *17*, 699–715.
3. Keglevich, Gy.; Grün, A.; Bölcskei, A.; Drahos, L.; Kraszni, M.; Balogh, Gy. T. *Heteroatom Chem.* **2012**, *23*, 574–582.
4. Takács-Novák, K.; Avdeef, A. *J. Pharm. Biomed. Anal.* **1996**, *14*, 1405–1413.
5. Avdeef, A. *Quant. Struct. Act. Relat.* **1992**, *11*, 510–517.
6. Hu, C.; Kitts, D. D. *Phytomed.* **2005**, *12*, 588–597.
7. Müller, J.; Esső, K.; Dargó, G.; Könczöl, Á.; Balogh, Gy. T. *Eur. J. Pharm. Sci.* **2015**, *79*, 53–60.
8. Pastan, I.; Gottesman, M. M.; Ueda, K.; Lovelace, E.; Rutherford, A. V.; Willingham M. C. *Proc. Nat. Acad. Sci. USA* **1988**, *85*, 4486–4490.
9. Martins, A.; Sipos, P.; Dér, K.; Csábi, J.; Miklos, W.; Berger, W.; Zalatnai, A.; Amaral, L.; Molnár, J.; Szabó-Révész, P.; Hunyadi, A. *Biomed. Res. Int.* **2015**, Article ID 895360.

10. MacroModel; Schrödinger, LLC, **2012**, http://www.schrodinger.com/MacroModel.

11. Frisch, M. J.; Trucks, G. W.; Schlegel, H. B.; Scuseria, G. E.; Robb, M. A.; Cheeseman, J. R.; Scalmani, G.; Barone, V.; Mennucci, B.; Petersson, G. A.; Nakatsuji, H.; Caricato, M.; Li, X.; Hratchian, H. P.; Izmaylov, A. F.; Bloino, J.; Zheng, G.; Sonnenberg, J. L.; Hada, M.; Ehara, M.; Toyota, K.; Fukuda, R.; Hasegawa, J.; Ishida, M.; Nakajima, T.; Honda, Y.; Kitao, O.; Nakai, H.; Vreven, T.; Montgomery, J. A., Jr.; Peralta, J. E.; Ogliaro, F.; Bearpark, M.; Heyd, J. J.; Brothers, E.; Kudin, K. N.; Staroverov, V. N.; Kobayashi, R.; Normand, J.; Raghavachari, K.; Rendell, A.; Burant, J. C.; Iyengar, S. S.; Tomasi, J.; Cossi, M.; Rega, N.; Millam, J. M.; Klene, M.; Knox, J. E.; Cross, J. B.; Bakken, V.; Adamo, C.; Jaramillo, J.; Gomperts, R.; Stratmann, R. E.; Yazyev, O.; Austin, A. J.; Cammi, R.; Pomelli, C.; Ochterski, J. W.; Martin, R. L.; Morokuma, K.; Zakrzewski, V. G.; Voth, G. A.; Salvador, P.; Dannenberg, J. J.; Dapprich, S.; Daniels, A. D.; Farkas, O.; Foresman, J. B.; Ortiz, J. V.; Cioslowski, J.; Fox, D. J. Gaussian 09, revision B.01; Gaussian, Inc.: Wallingford, CT, **2010**.

12. Stephens, P. J.; Harada, N. *Chirality* **2010**, *22*, 229–233.

13. Varetto, U. MOLEKEL, v. 5.4; Swiss National Supercomputing Centre: Manno, Switzerland, **2009**.

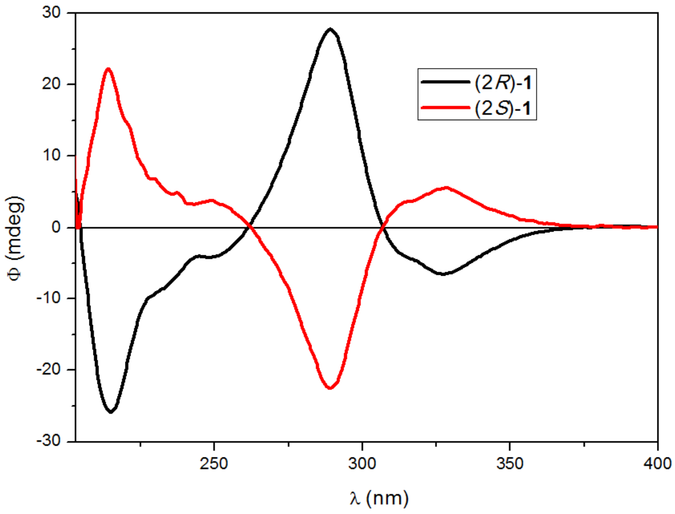

Figure S1: HPLC–ECD spectra of (2*R*)- and (2*S*)-naringenin (**1**).

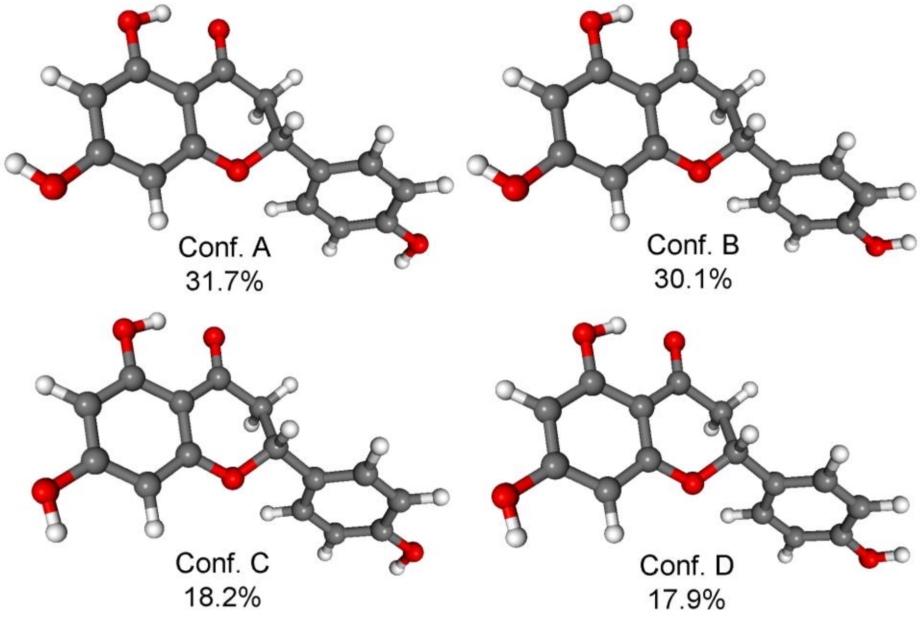

Figure S2: Structure and population of the low-energy B3LYP/6-31G(d) in vacuo conformers (>2%) of (*R*)-**1**.

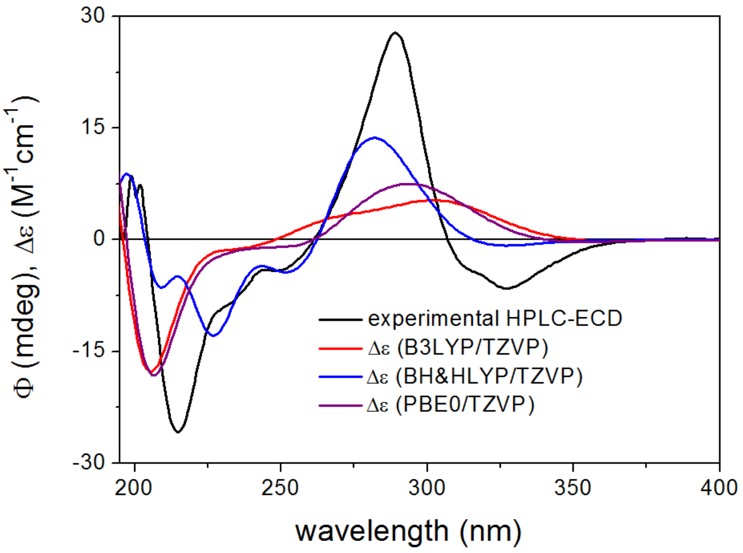

Figure S3: Experimental HPLC–ECD spectrum of (*R*)-**1** compared with the Boltzmann-weighted ECD spectra computed for the B3LYP/6-31G(d) in vacuo low-energy conformers at various levels.

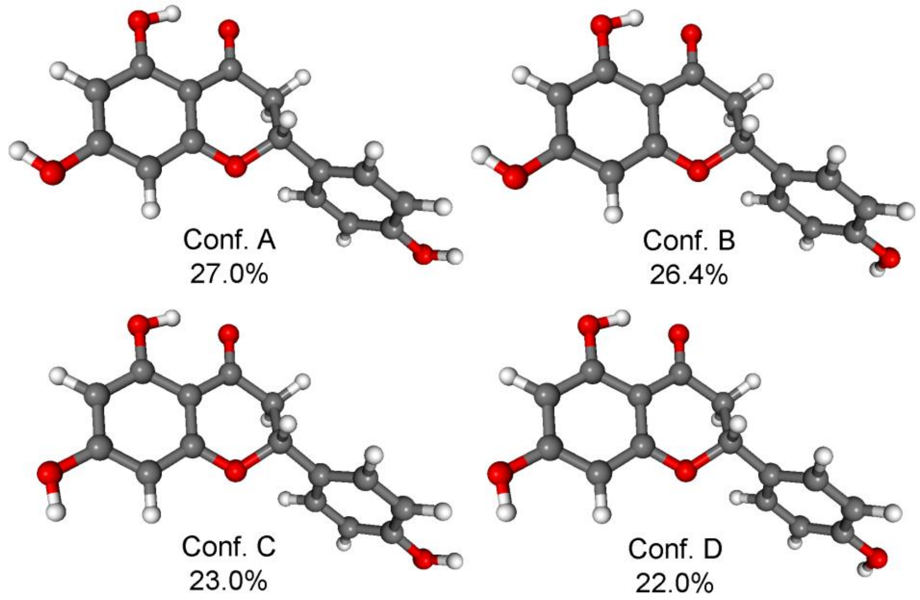

Figure S4: Structure and population of the low-energy B3LYP/TZVP PCM/$CHCl_3$ conformers (>2%) of (*R*)-**1**.

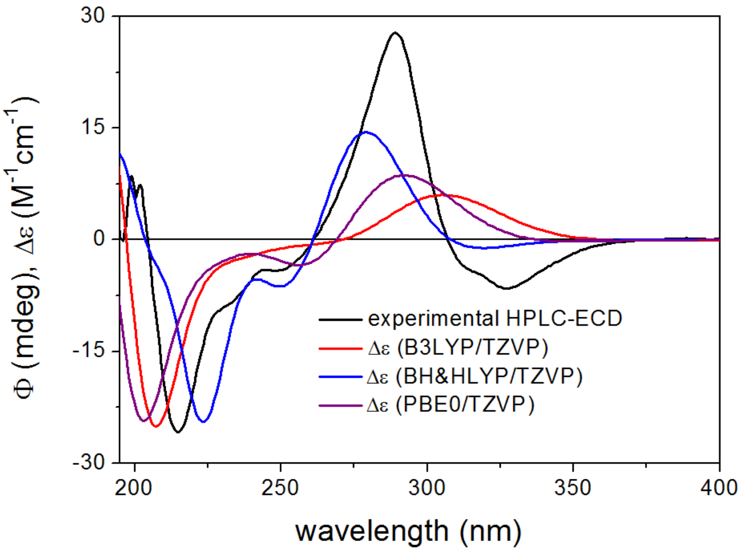

Figure S5: Experimental HPLC–ECD spectrum of (*R*)-**1** compared with the Boltzmann-weighted ECD spectra computed for the B3LYP/TZVP PCM/$CHCl_3$ low-energy conformers at various levels.

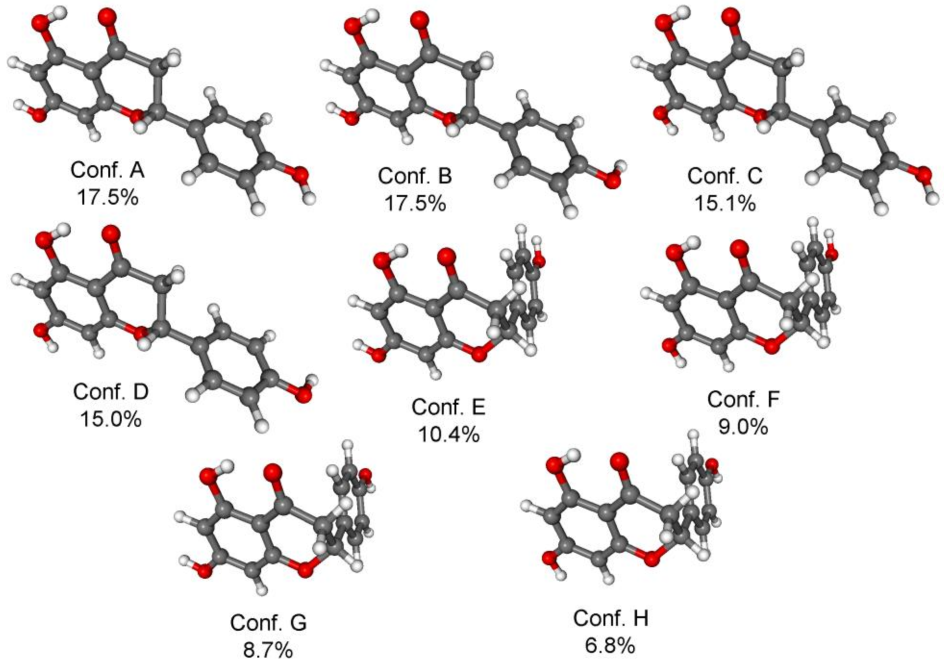

Figure S6: Structure and population of the low-energy B97D/TZVP PCM/$CHCl_3$ conformers (>2%) of (*R*)-**1**.

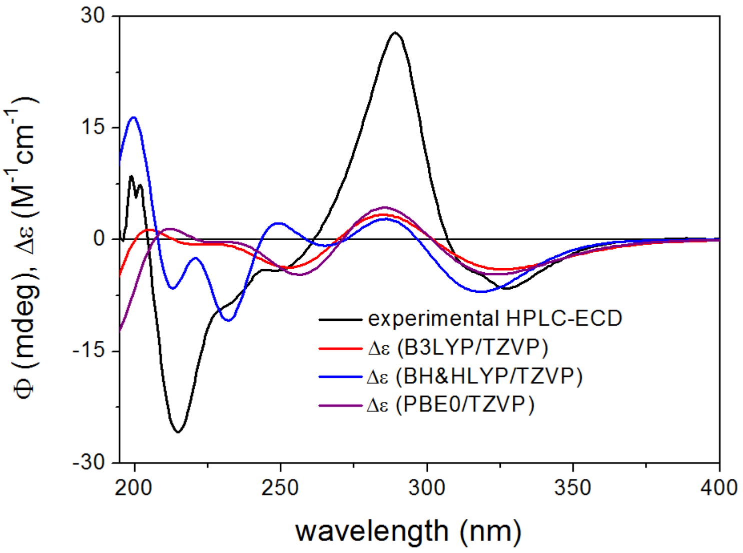

Figure S7: Experimental HPLC–ECD spectrum of (*R*)-**1** compared with the Boltzmann-weighted ECD spectra computed for the B97D/TZVP PCM/$CHCl_3$ low-energy conformers at various levels.

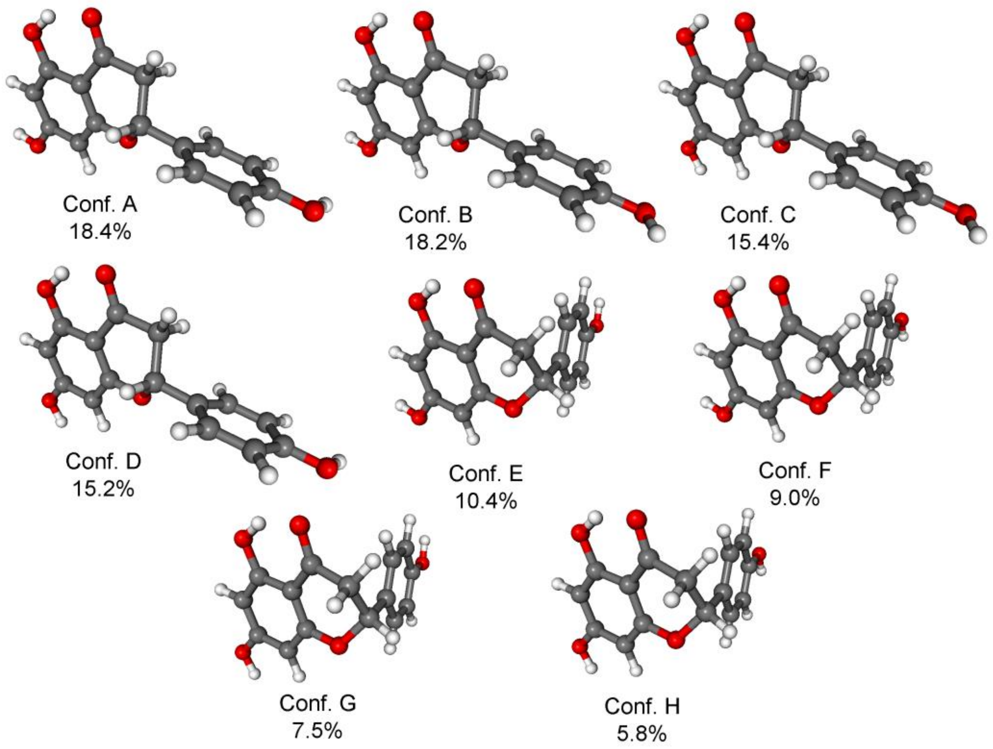

Figure S8: Structure and population of the low-energy M06-2X/TZVP PCM/$CHCl_3$ conformers (>2%) of (*R*)-**1**.

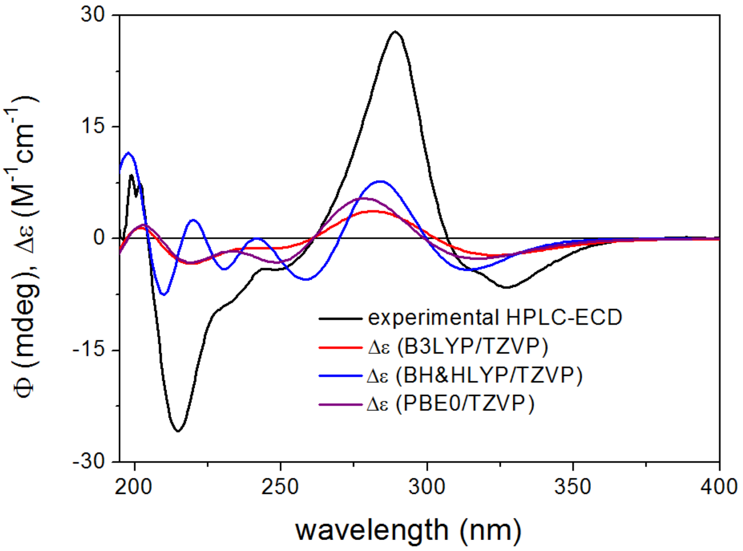

Figure S9: Experimental HPLC–ECD spectrum of (*R*)-**1** compared with the Boltzmann-weighted ECD spectra computed for the M06-2X/TZVP PCM/$CHCl_3$ low-energy conformers at various levels.

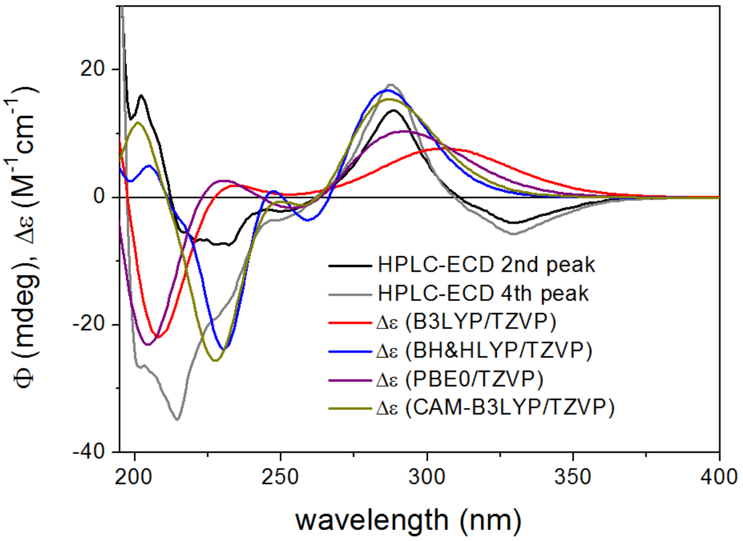

Figure S10: Experimental HPLC–ECD spectra of **2a** and **2d** compared with the Boltzmann-weighted ECD spectra computed for the CAM-B3LYP/TZVP PCM/MeCN low-energy conformers of (2*R*,5’’*R*)-**2** at various levels.

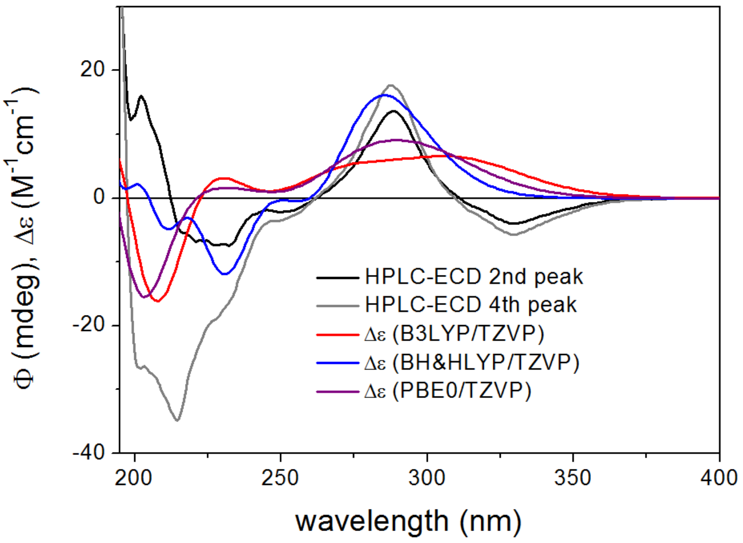

Figure S11: Experimental HPLC–ECD spectra of **2a** and **2d** compared with the Boltzmann-weighted ECD spectra computed for the B3LYP/6-31G(d) in vacuo low-energy conformers of (2*R*,5’’*R*)-**2** at various levels.

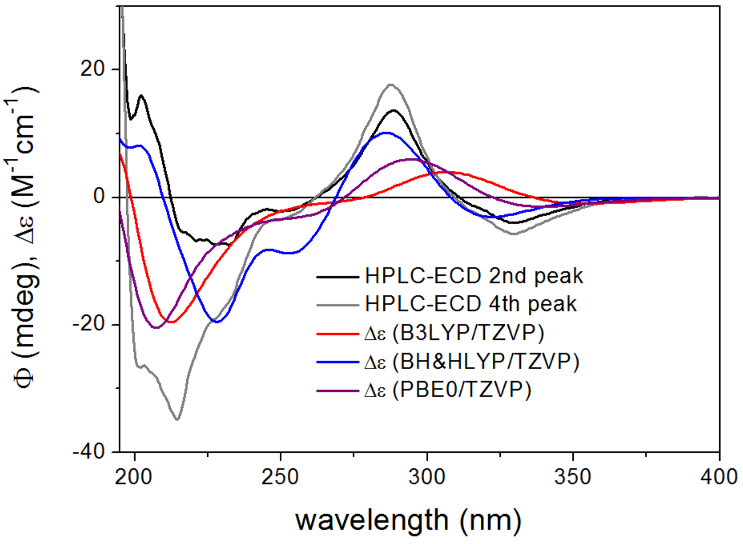

Figure S12: Experimental HPLC–ECD spectra of **2a** and **2d** compared with the Boltzmann-weighted ECD spectra computed for the B3LYP/6-31G(d) in vacuo low-energy conformers of (2*R*,5’’*S*)-**2** at various levels.

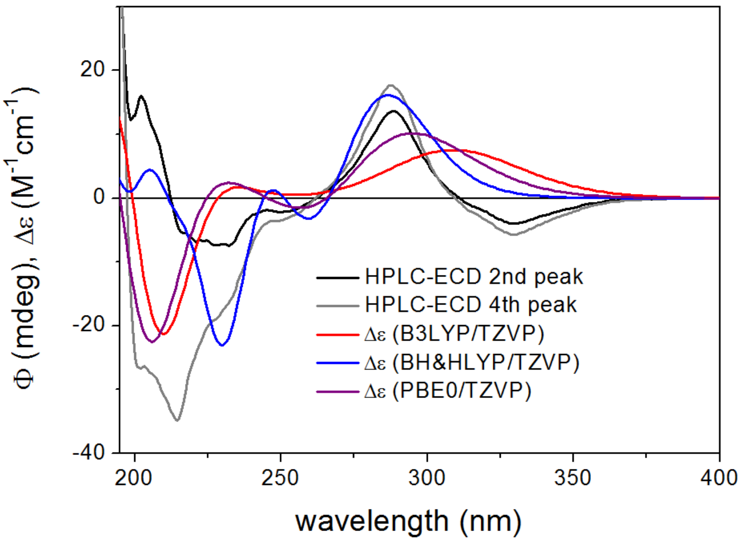

Figure S13: Experimental HPLC–ECD spectra of **2a** and **2d** compared with the Boltzmann-weighted ECD spectra computed for the B3LYP/TZVP PCM/MeCN low-energy conformers of (2*R*,5’’*R*)-**2** at various levels.

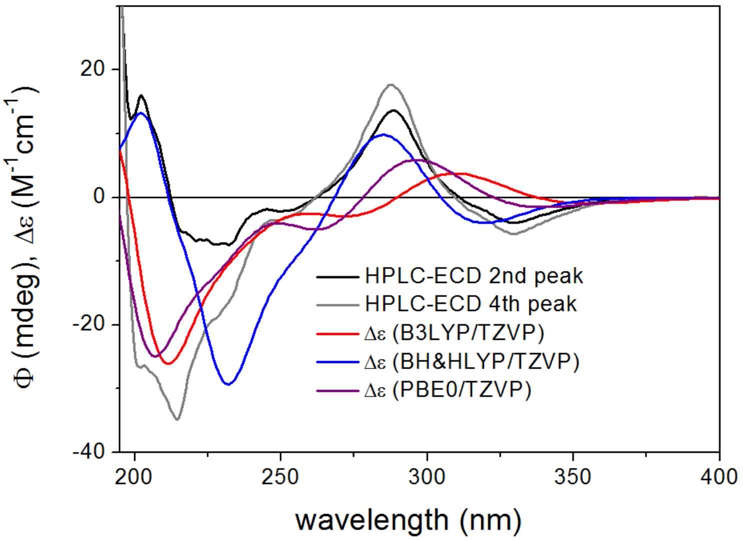

Figure S14: Experimental HPLC–ECD spectra of **2a** and **2d** compared with the Boltzmann-weighted ECD spectra computed for the B3LYP/TZVP PCM/MeCN low-energy conformers of (2*R*,5’’*S*)-**2** at various levels.

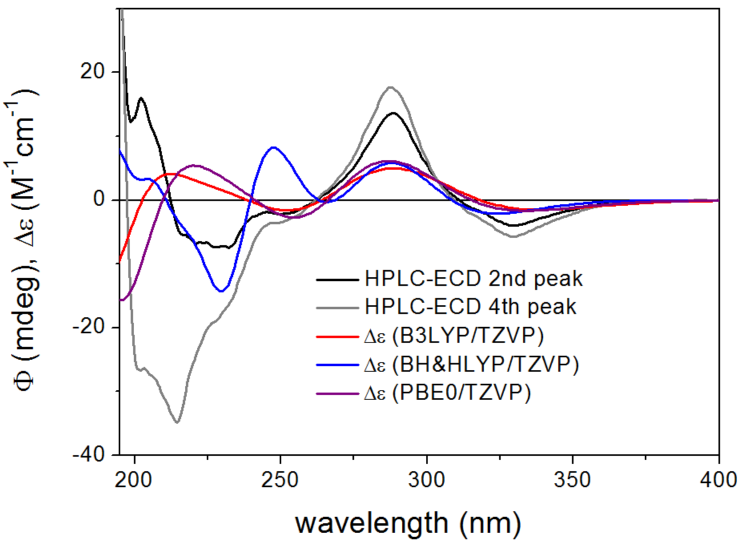

Figure S15: Experimental HPLC–ECD spectra of **2a** and **2d** compared with the Boltzmann-weighted ECD spectra computed for the B97D/TZVP PCM/MeCN low-energy conformers of (2*R*,5’’*R*)-**2** at various levels.

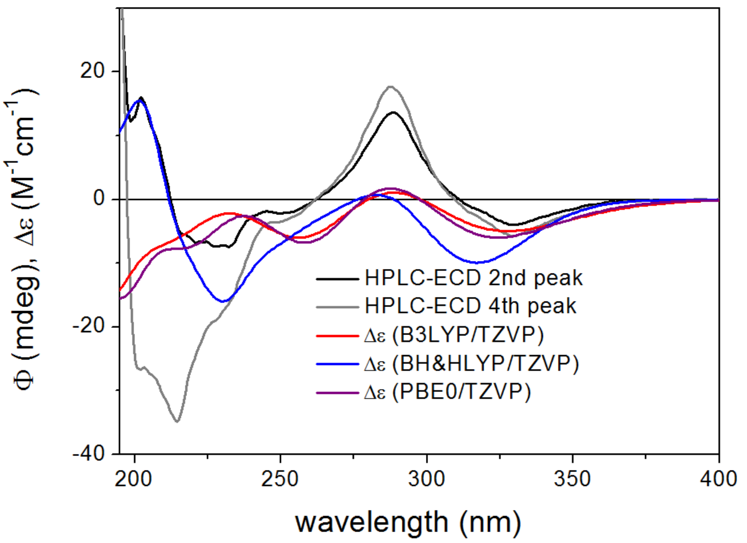

Figure S16: Experimental HPLC–ECD spectra of **2a** and **2d** compared with the Boltzmann-weighted ECD spectra computed for the B97D/TZVP PCM/MeCN low-energy conformers of (2*R*,5’’*S*)-**2** at various levels.

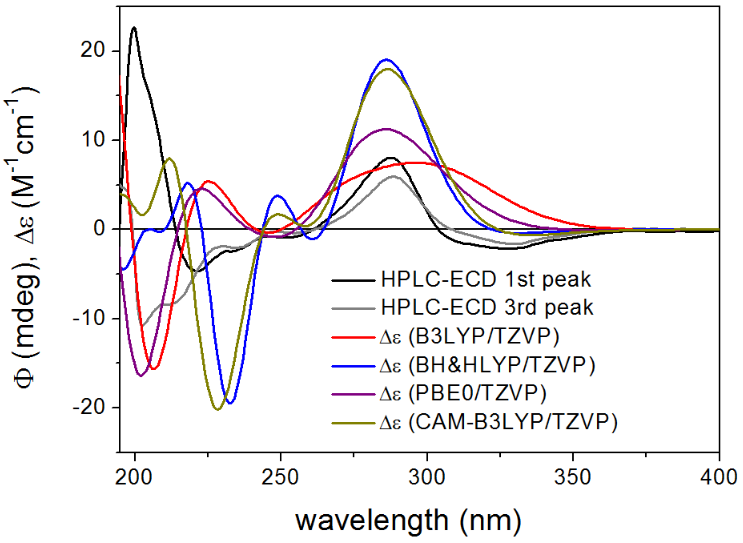

Figure S17: Experimental HPLC–ECD spectra of **3b** and **3c** compared with the Boltzmann-weighted ECD spectra computed for the CAM-B3LYP/TZVP PCM/MeCN low-energy conformers of (2*R*,5’’*R*)-**3** at various levels.

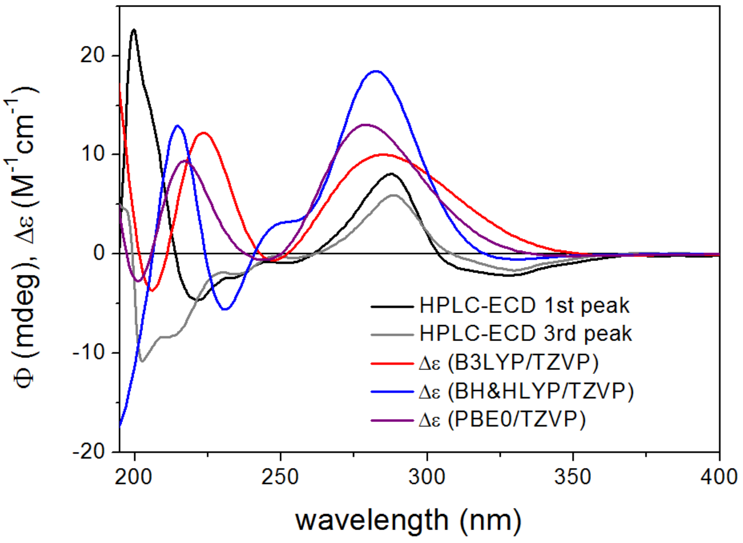

Figure S18: Experimental HPLC–ECD spectra of **3b** and **3c** compared with the Boltzmann-weighted ECD spectra computed for the B3LYP/6-31G(d) in vacuo low-energy conformers of (2*R*,5’’*R*)-**3** at various levels.

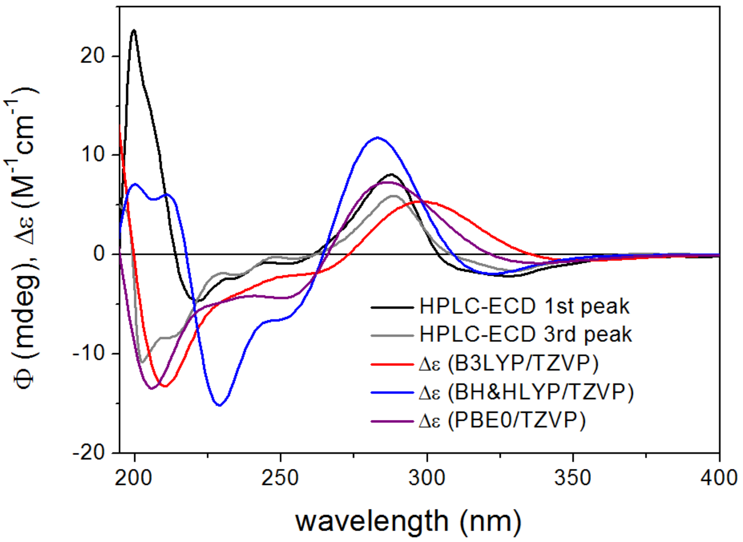

Figure S19: Experimental HPLC–ECD spectra of **3b** and **3c** compared with the Boltzmann-weighted ECD spectra computed for the B3LYP/6-31G(d) in vacuo low-energy conformers of (2*R*,5’’*S*)-**3** at various levels.

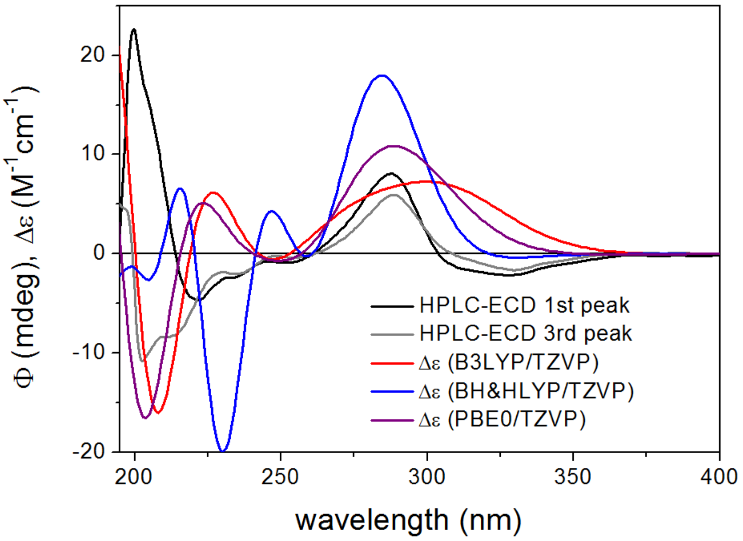

Figure S20: Experimental HPLC–ECD spectra of **3b** and **3c** compared with the Boltzmann-weighted ECD spectra computed for the B3LYP/TZVP PCM/MeCN low-energy conformers of (2*R*,5’’*R*)-**3** at various levels.

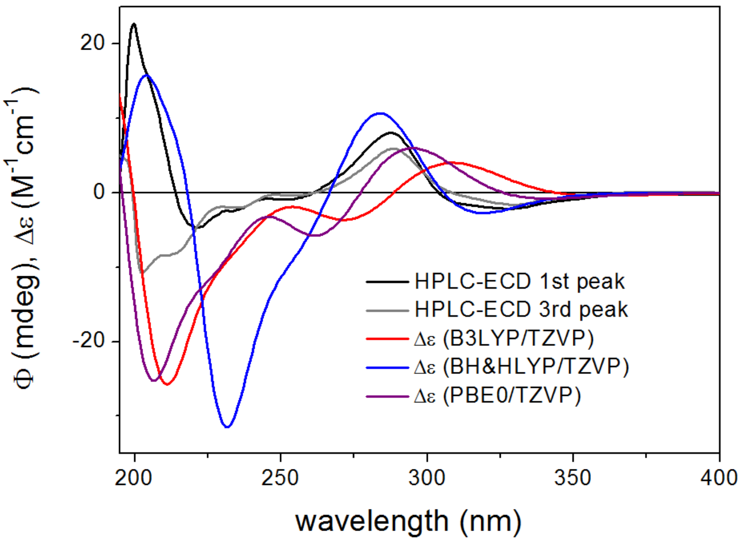

Figure S21: Experimental HPLC–ECD spectra of **3b** and **3c** compared with the Boltzmann-weighted ECD spectra computed for the B3LYP/TZVP PCM/MeCN low-energy conformers of (2*R*,5’’*S*)-**3** at various levels.

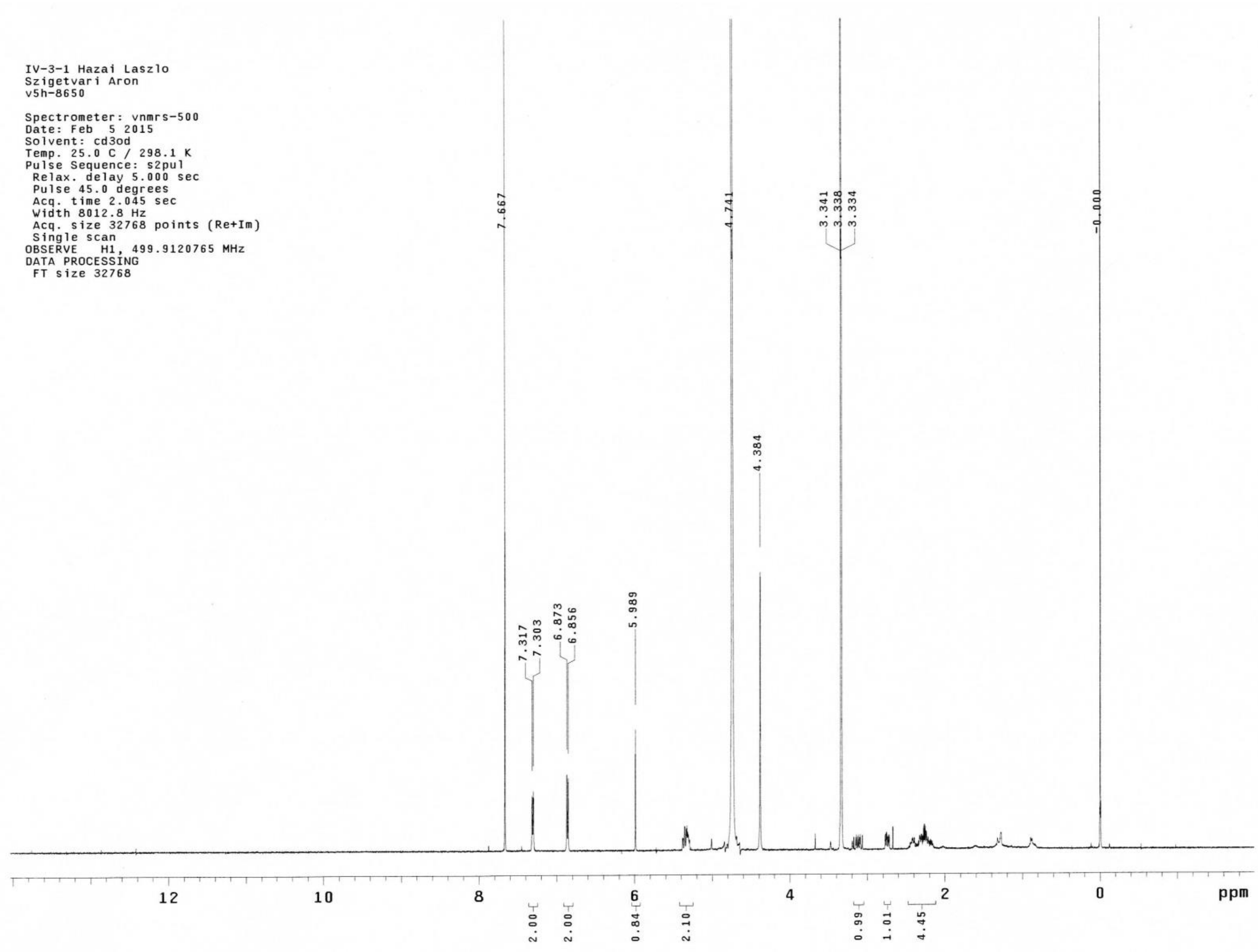

Figure S22: ^{1}H NMR spectrum of **(±)-3a–d** in CD_3OD.

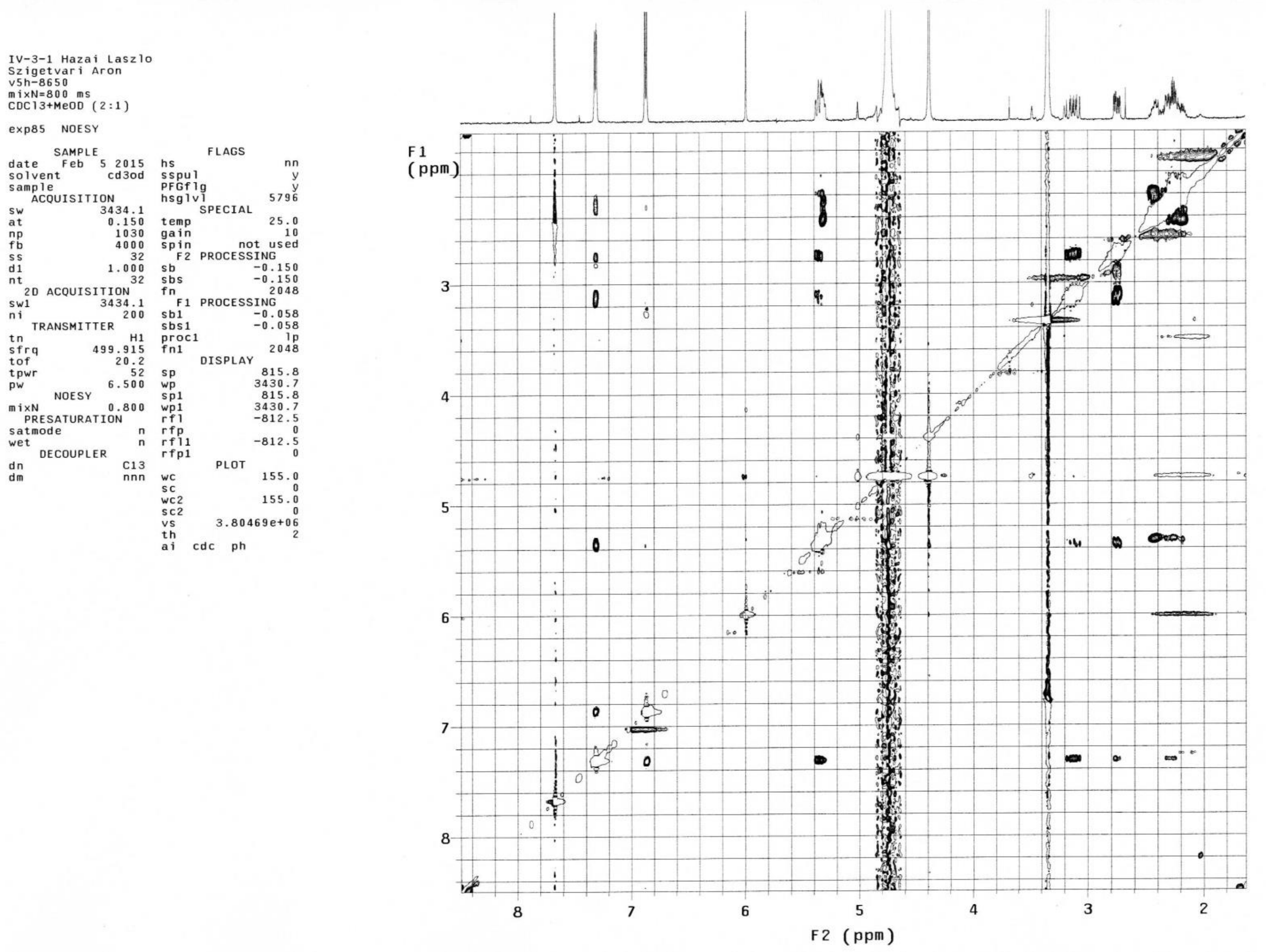

Figure S23: NOESY spectrum of **(±)-3a–d** (No. 1).

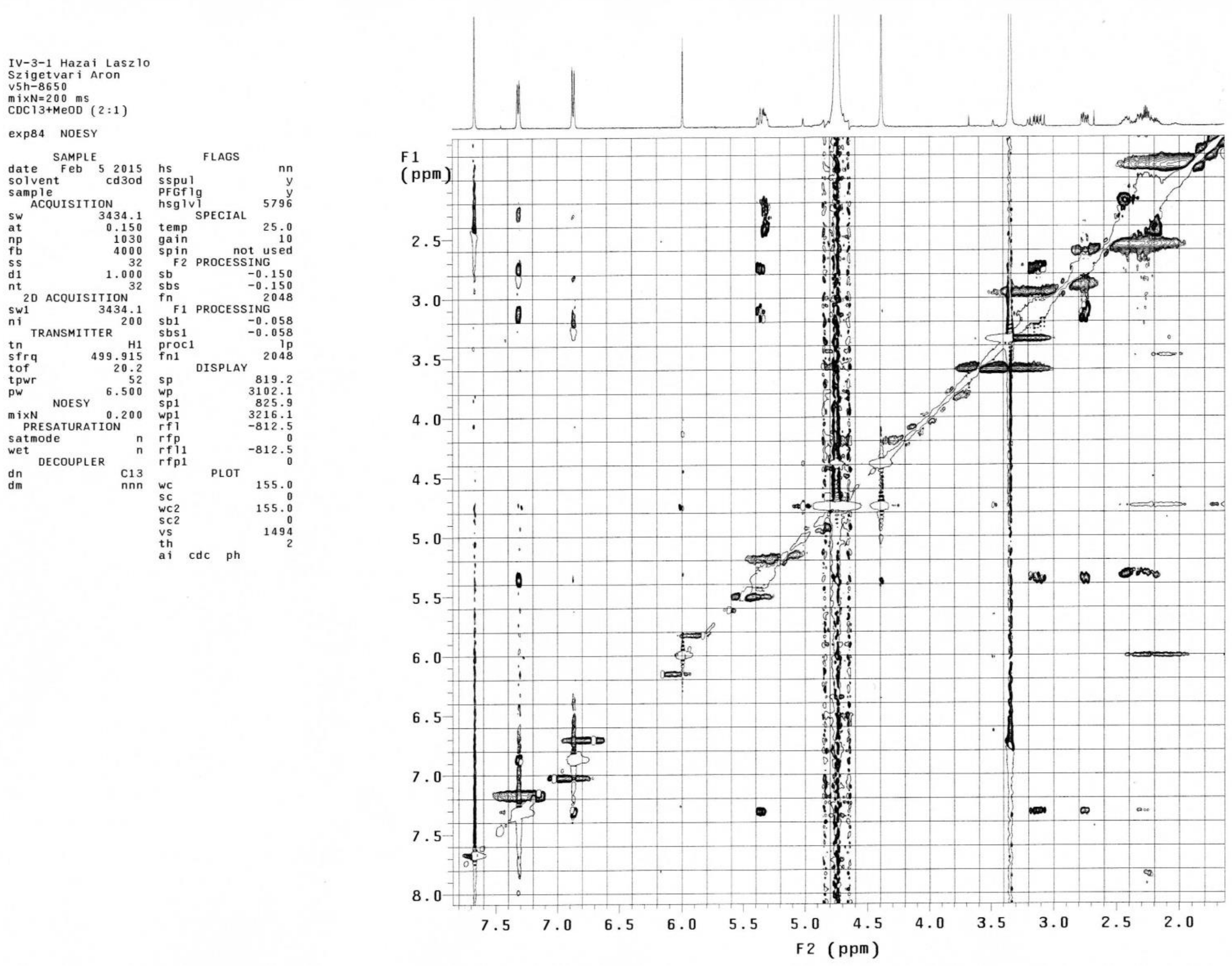

Figure S24: NOESY spectrum of **(±)-3a–d** (No. 2).

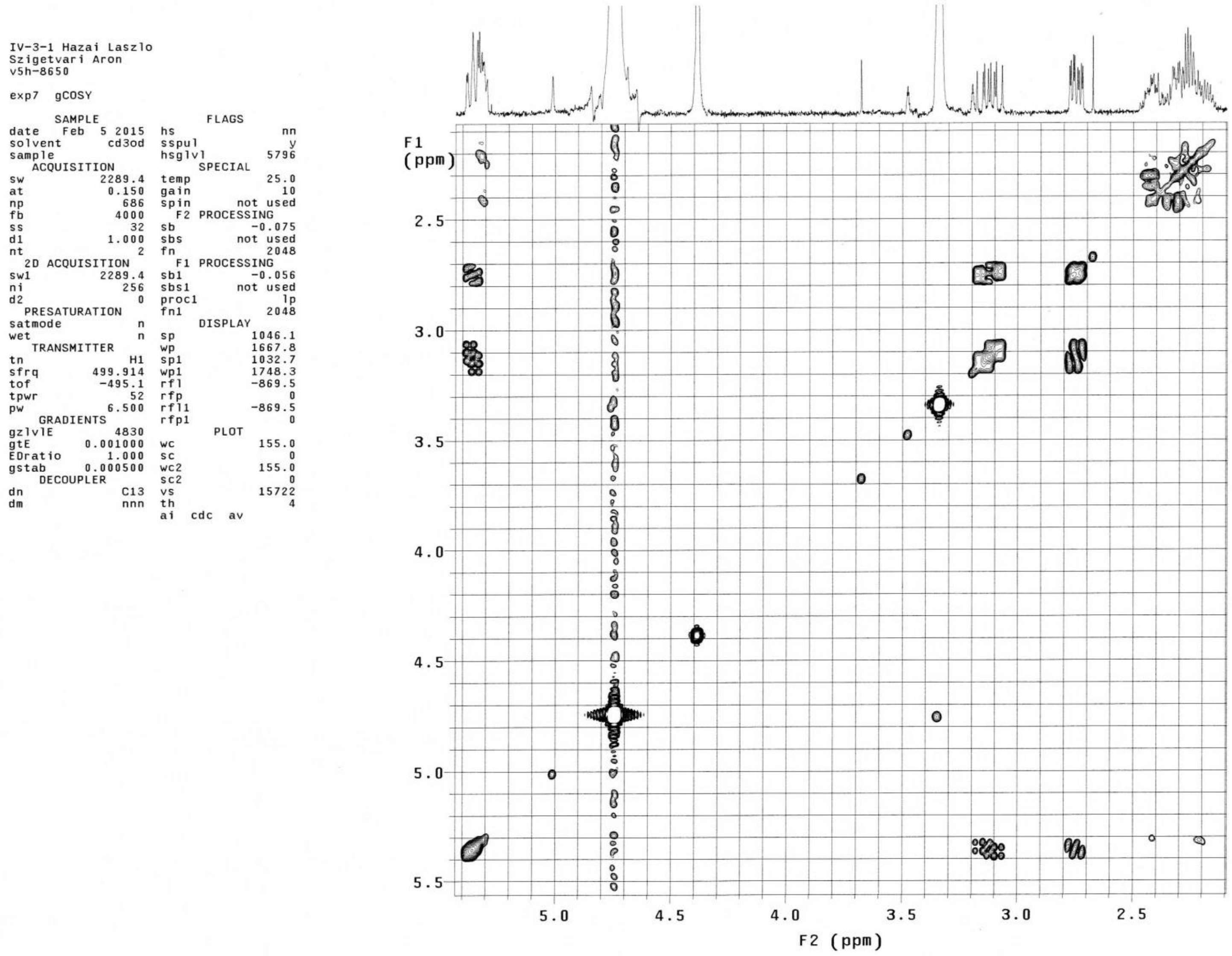

Figure S25: gCOSY spectrum of **(±)-3a–d**.

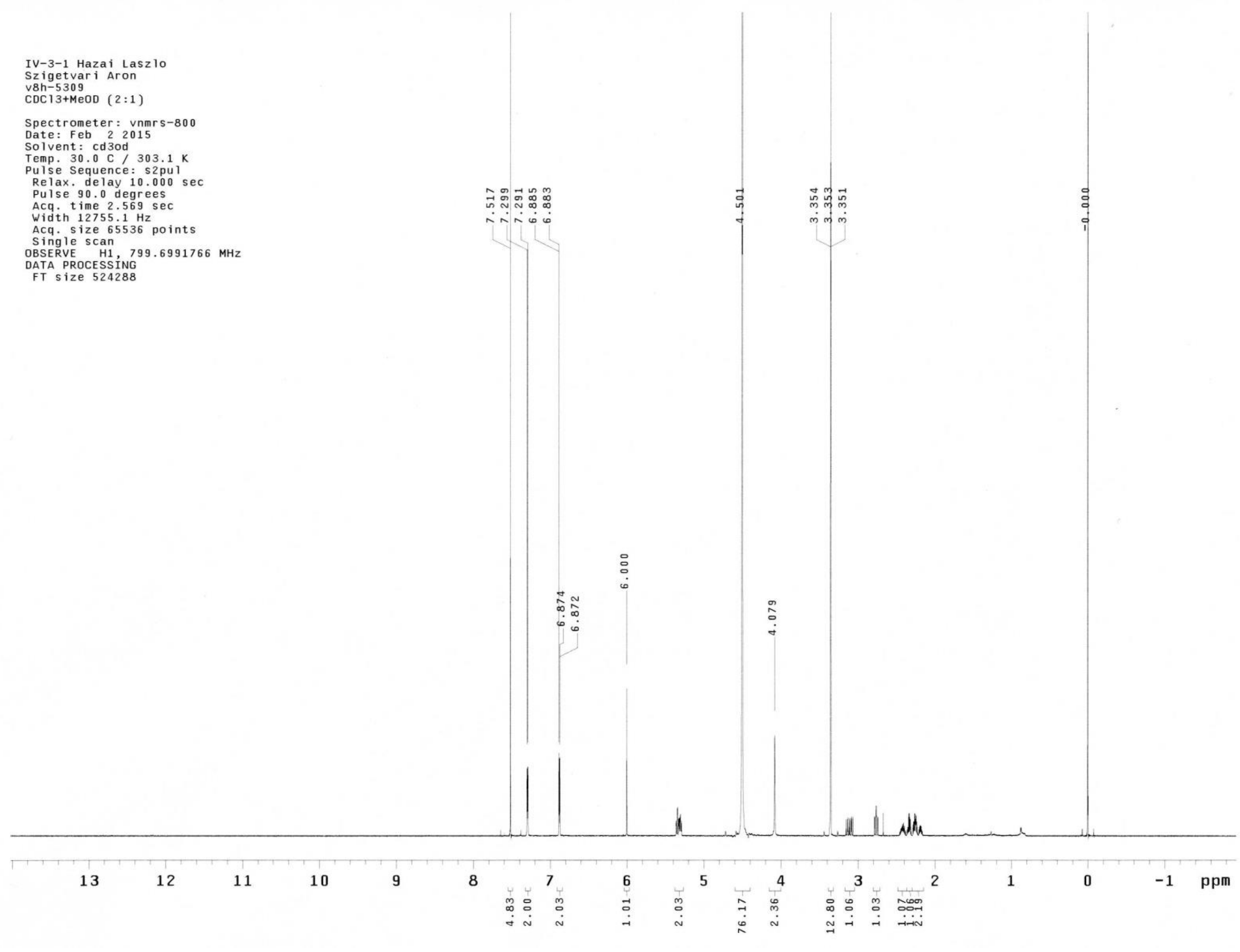

Figure S26: ^{1}H NMR spectrum of **(±)-3a–d** in $CDCl_3$: CD_3OD (2:1).

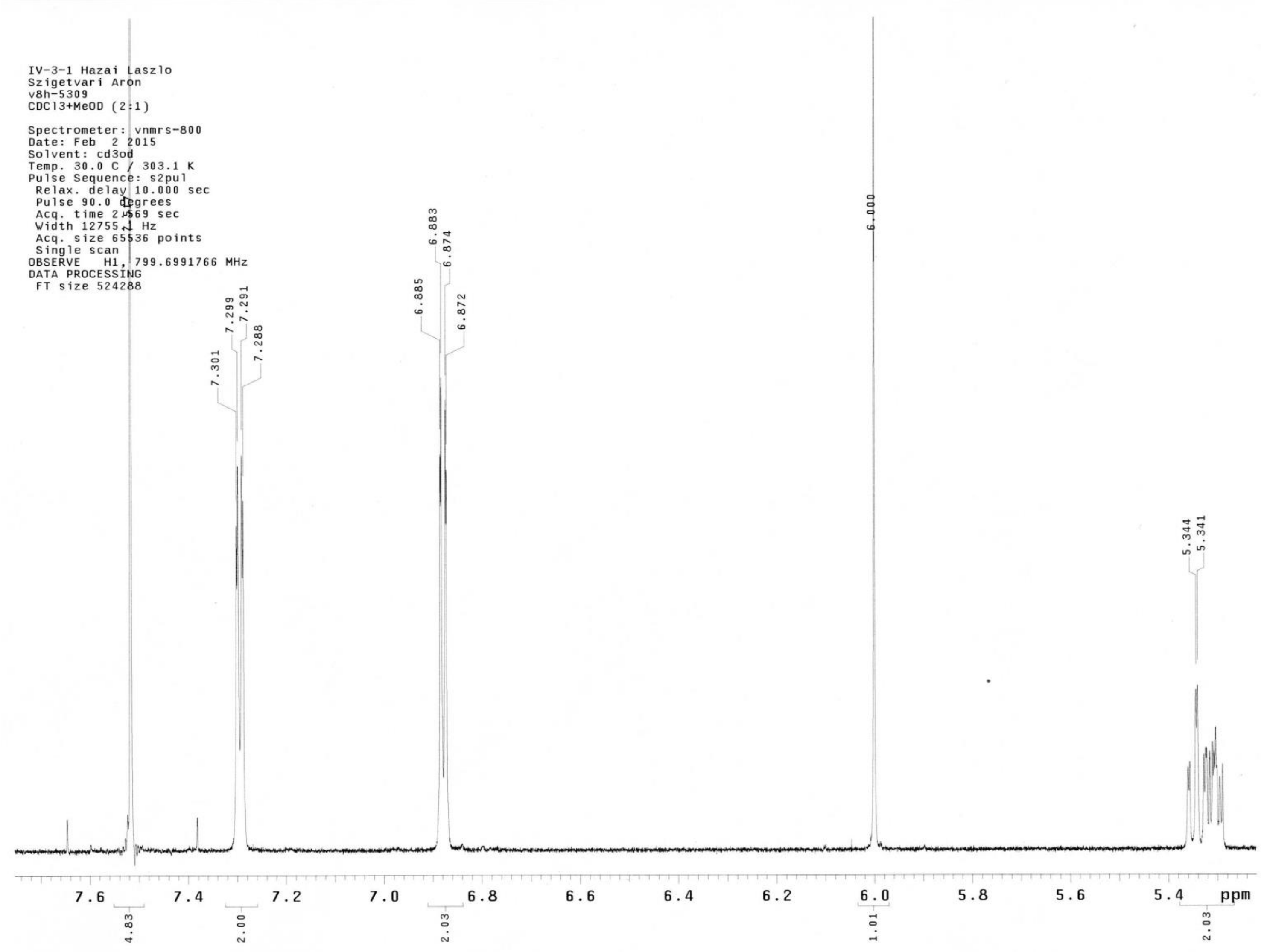

Figure S27: ^{1}H NMR spectrum of **(±)-3a–d** in $CDCl_3$: CD_3OD (2:1) – zoom from 5.24 to 7.74 ppm.

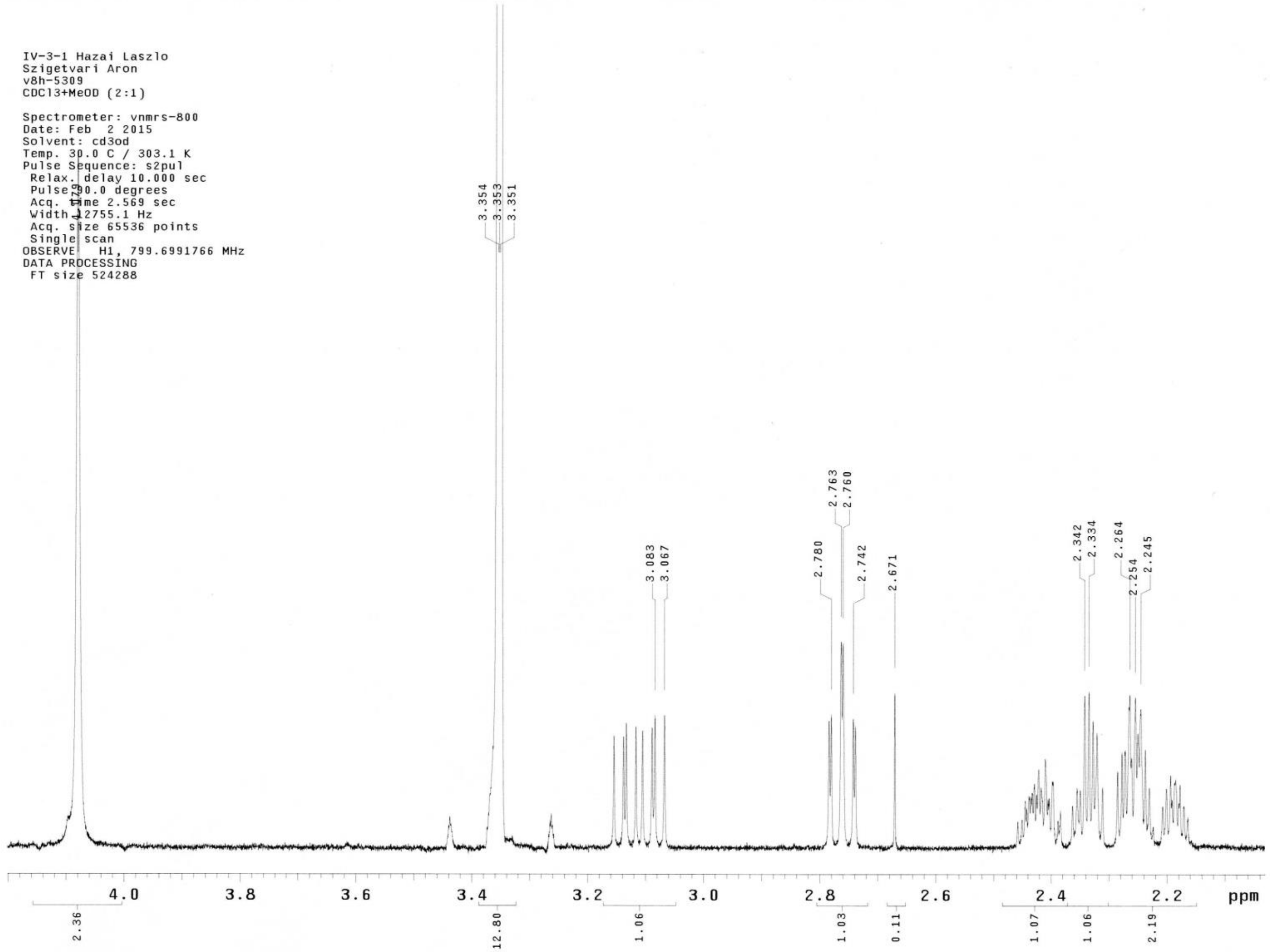

Figure S28: ^{1}H NMR spectrum of **(±)-3a–d** in $CDCl_3$: CD_3OD (2:1) – zoom from 2.04 to 4.20 ppm.

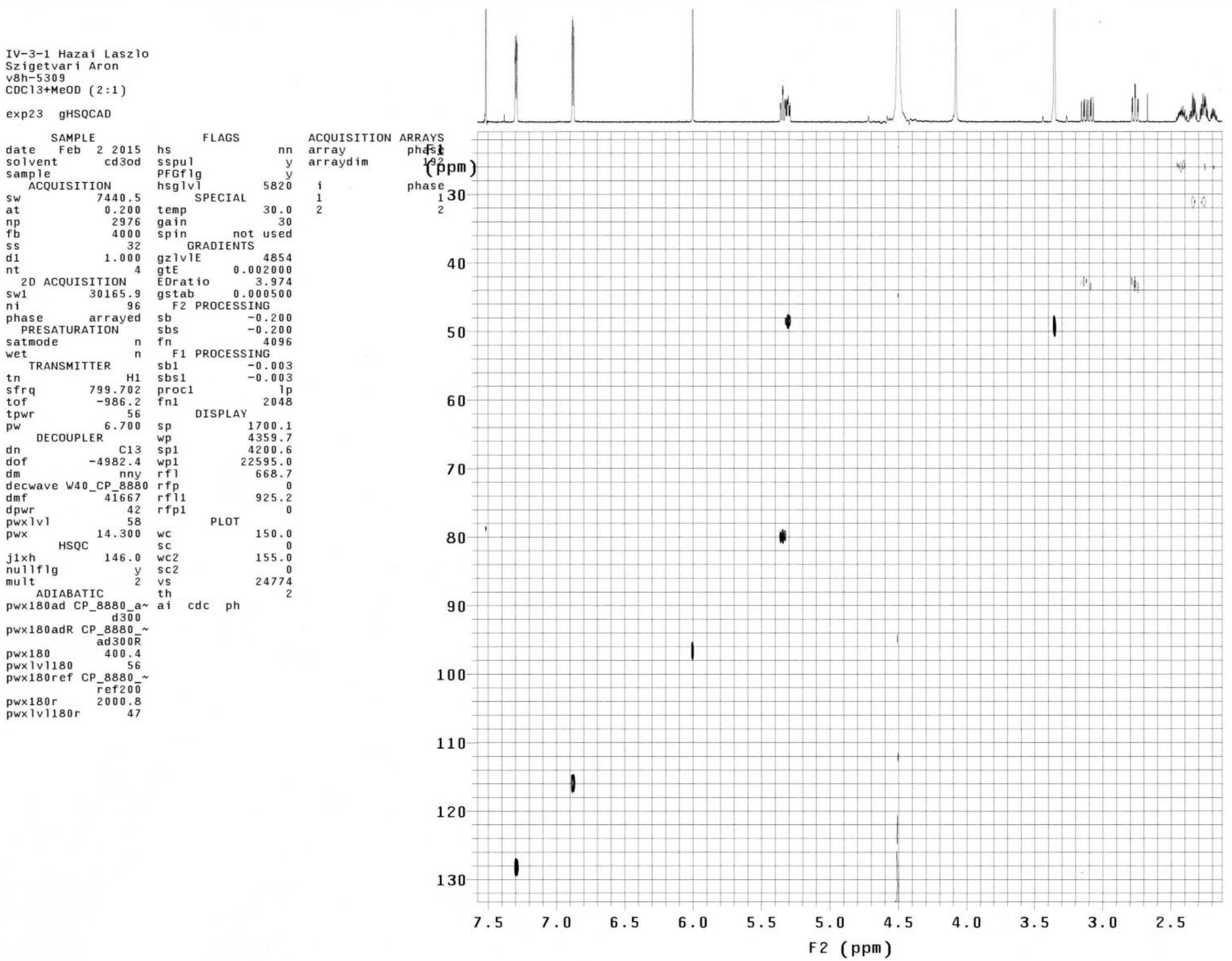

Figure S29: gHSQCad spectrum of **(±)-3a–d**.

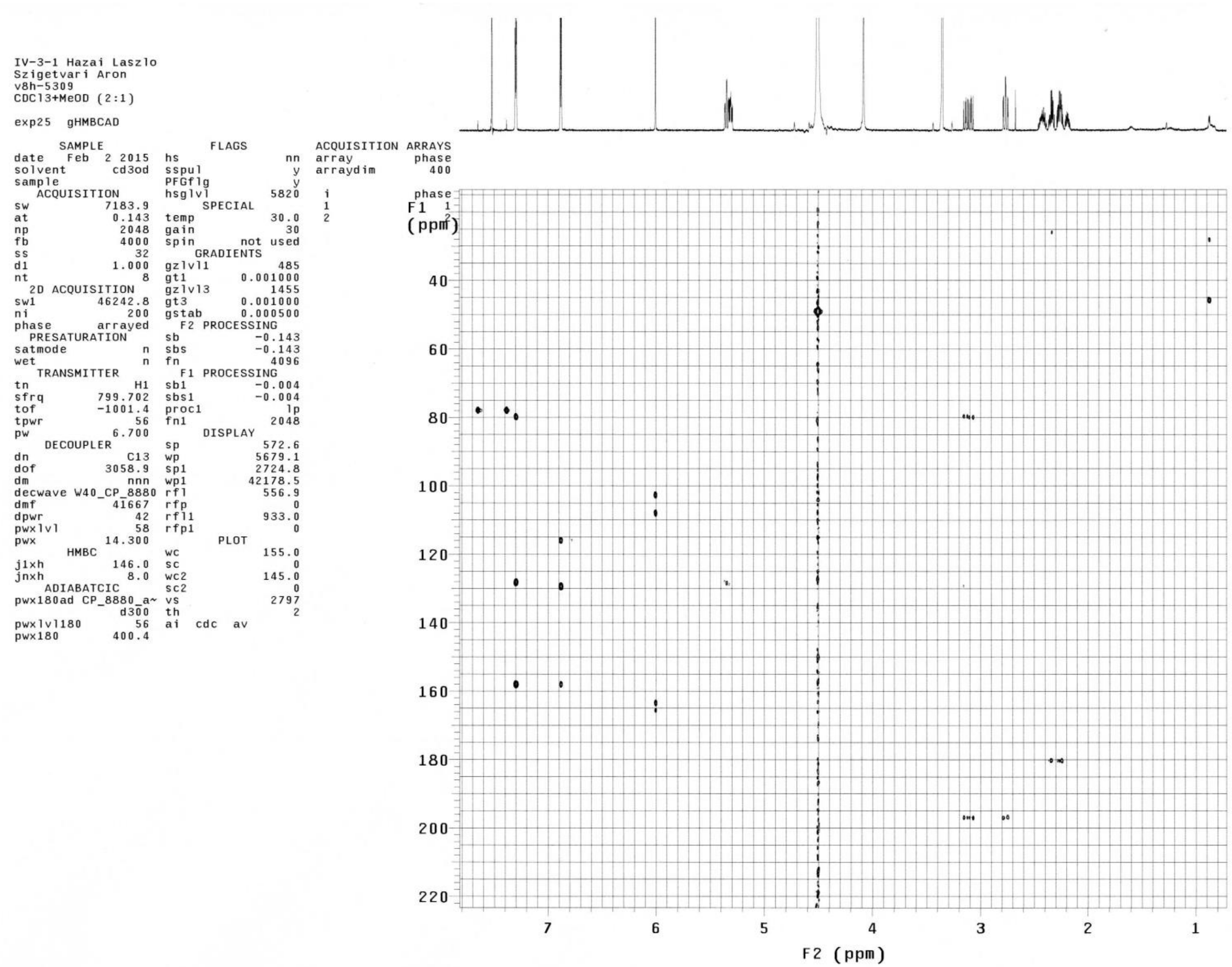

Figure S30: gHMBCad spectrum of **(±)-3a–d**.

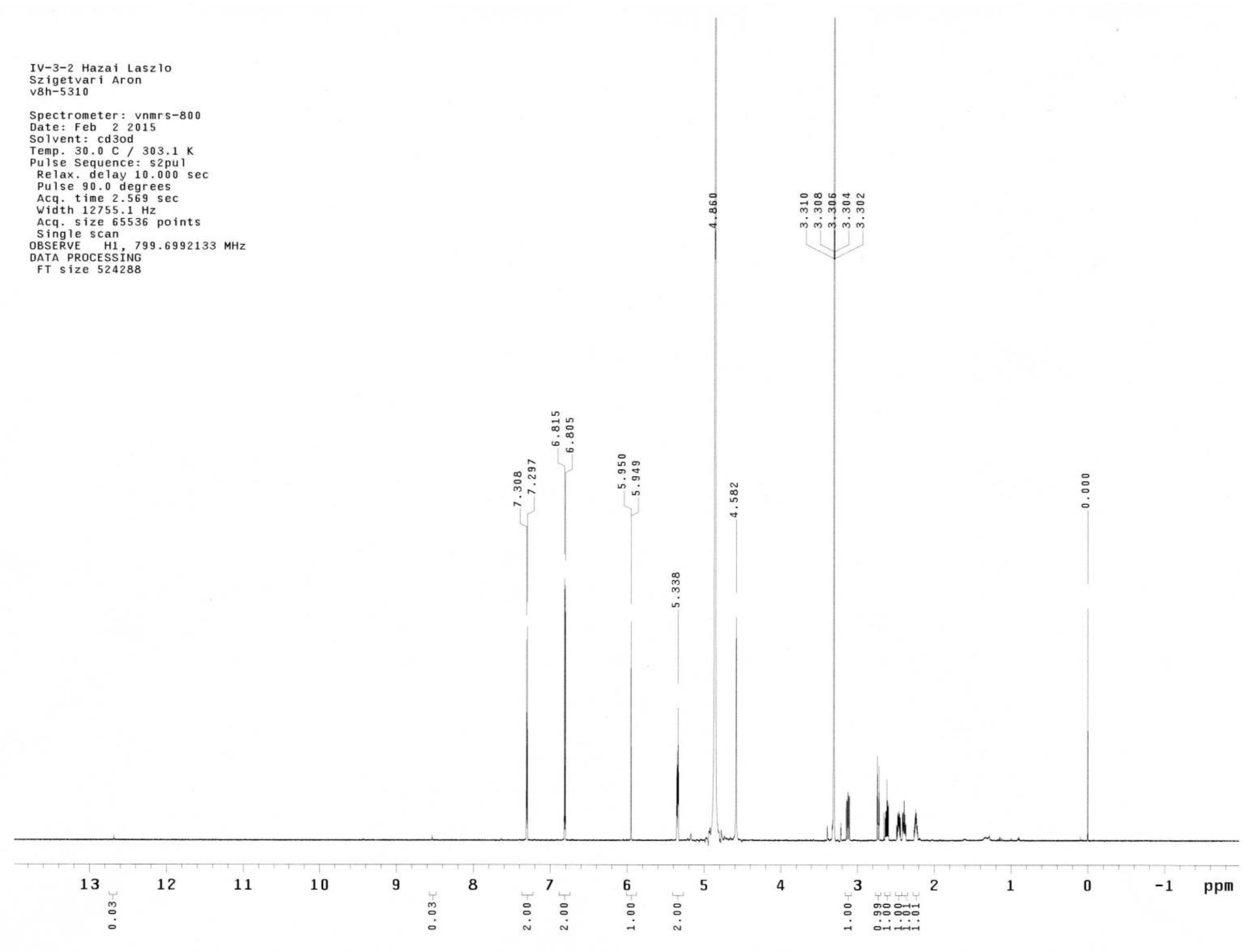

Figure S31: ^{1}H NMR spectrum of **(±)-2a–d**.

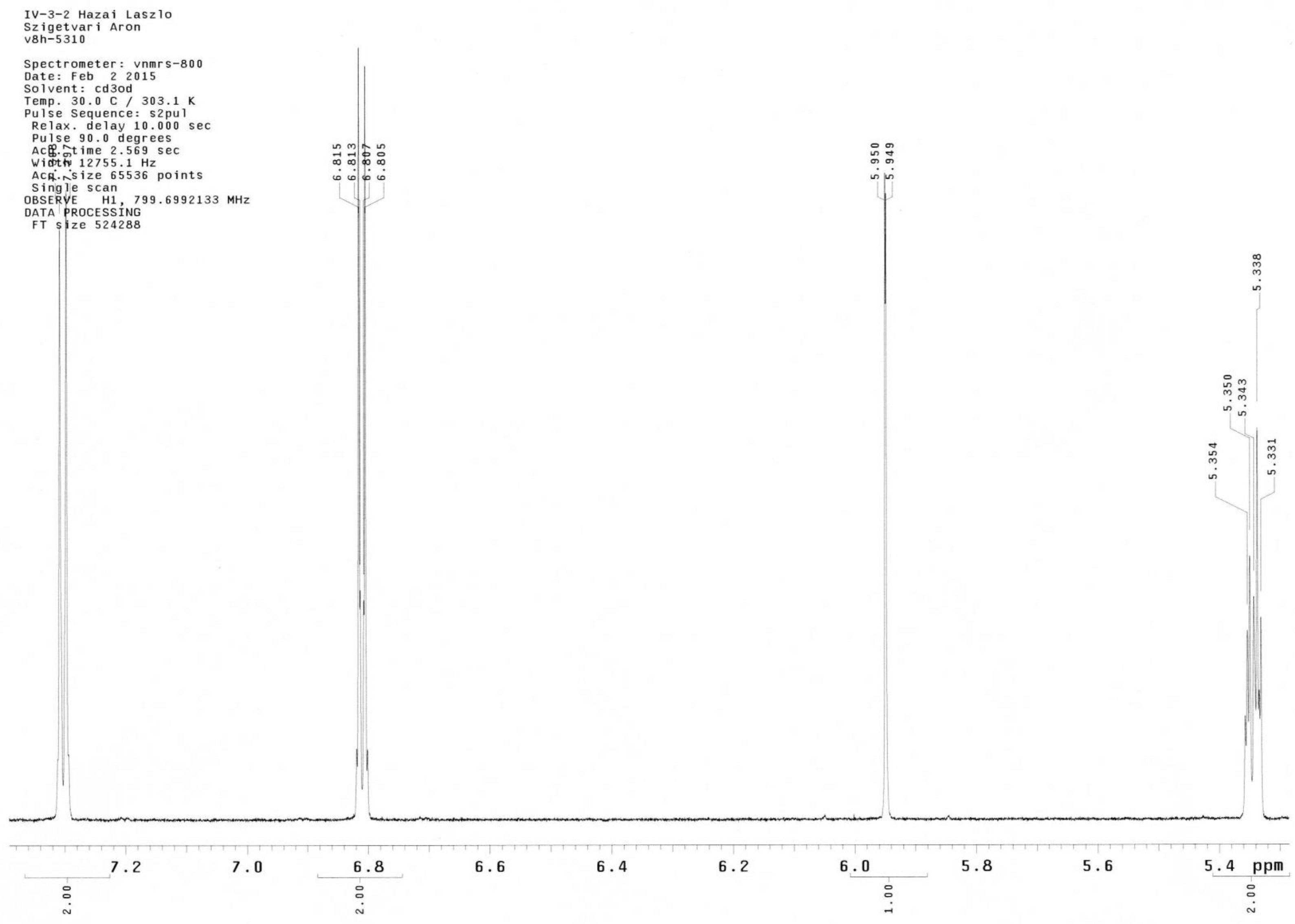

Figure S32: ^{1}H NMR spectrum of **(±)-2a–d** – zoom from 5.30 to 7.38 ppm.

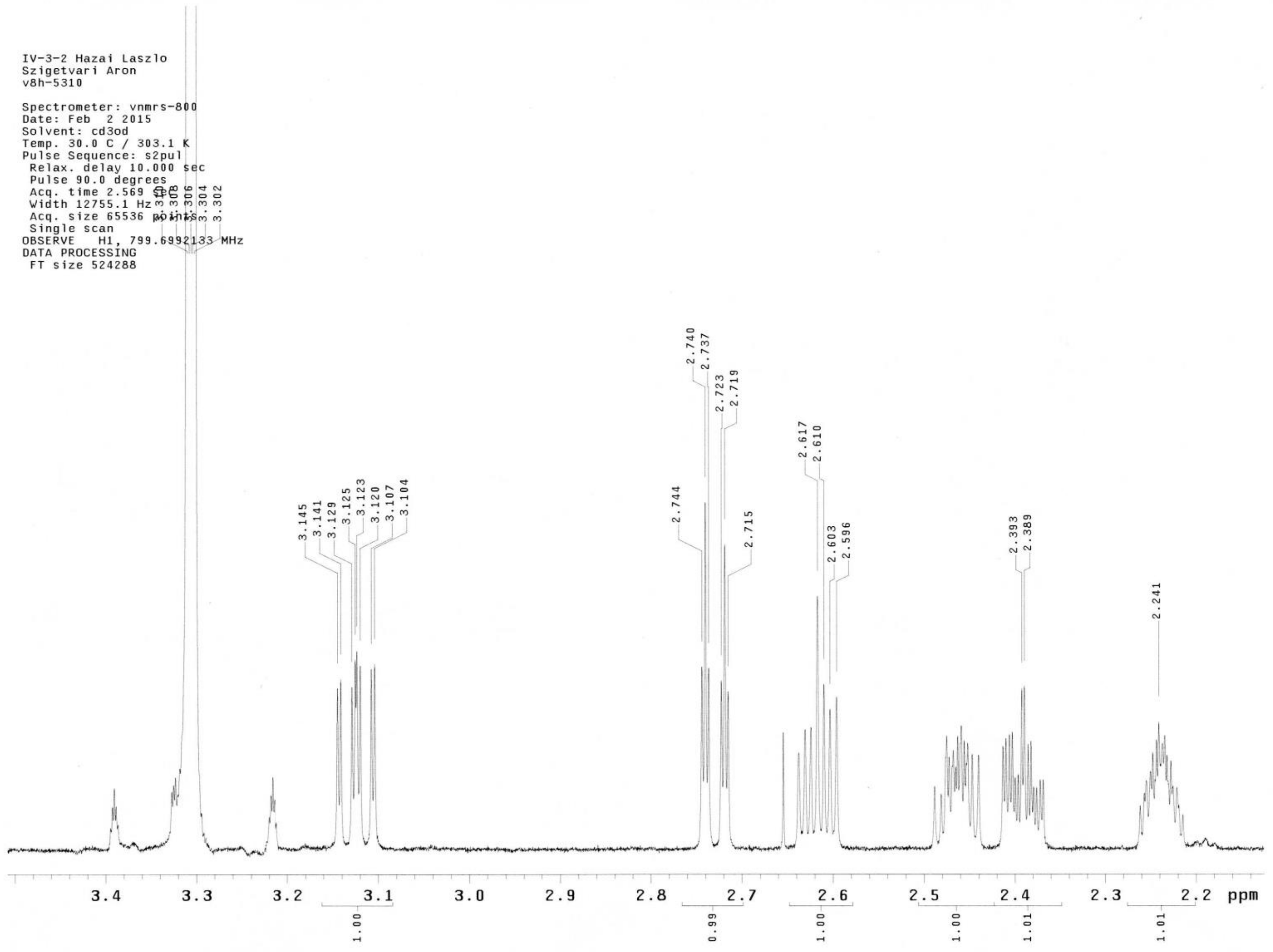

Figure S33: ^{1}H NMR spectrum of **(±)-2a–d** – zoom from 2.14 to 3.50 ppm.

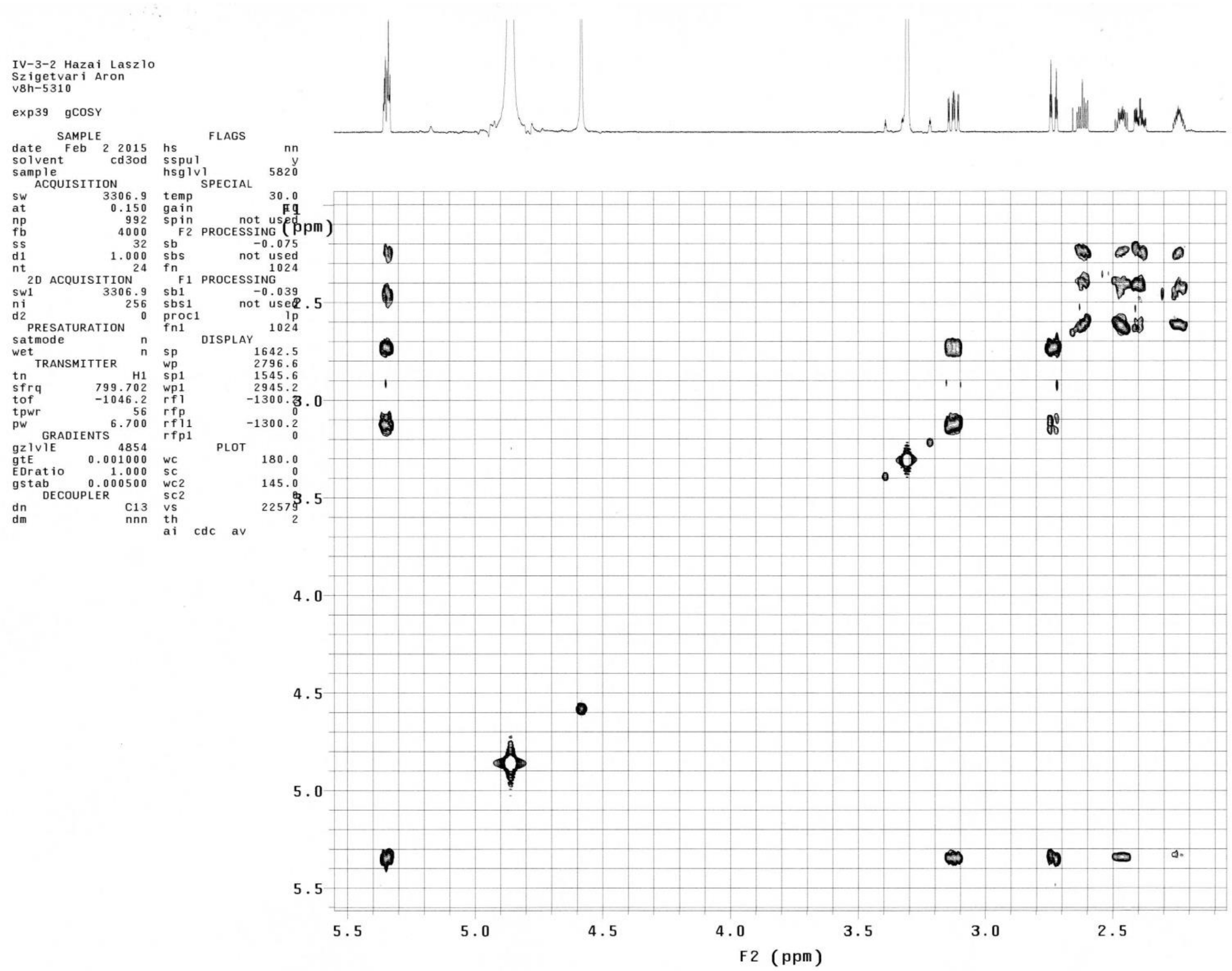

Figure S34: gCOSY spectrum of **(±)-2a–d**.

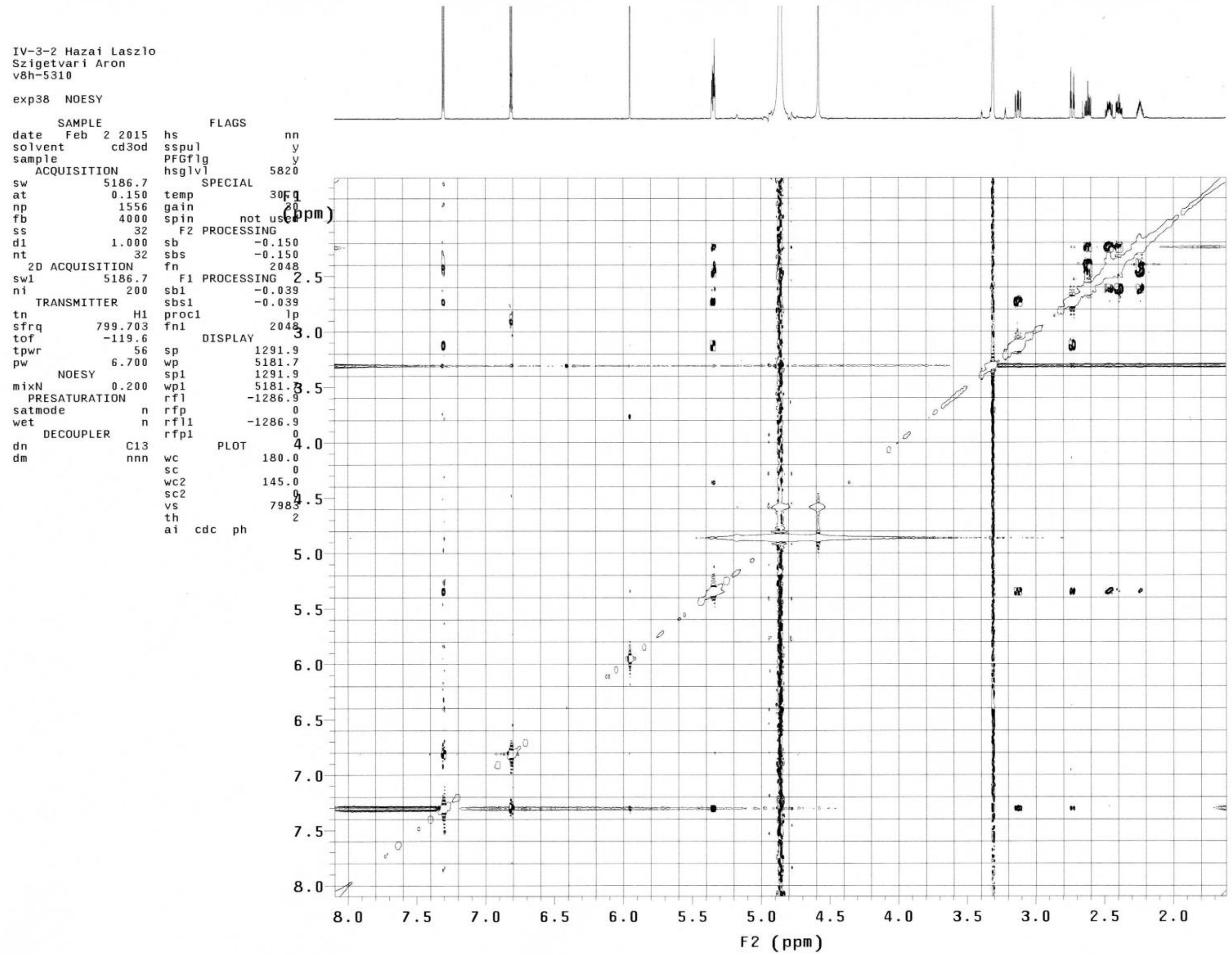

Figure S35: NOESY spectrum of **(±)-2a–d**.

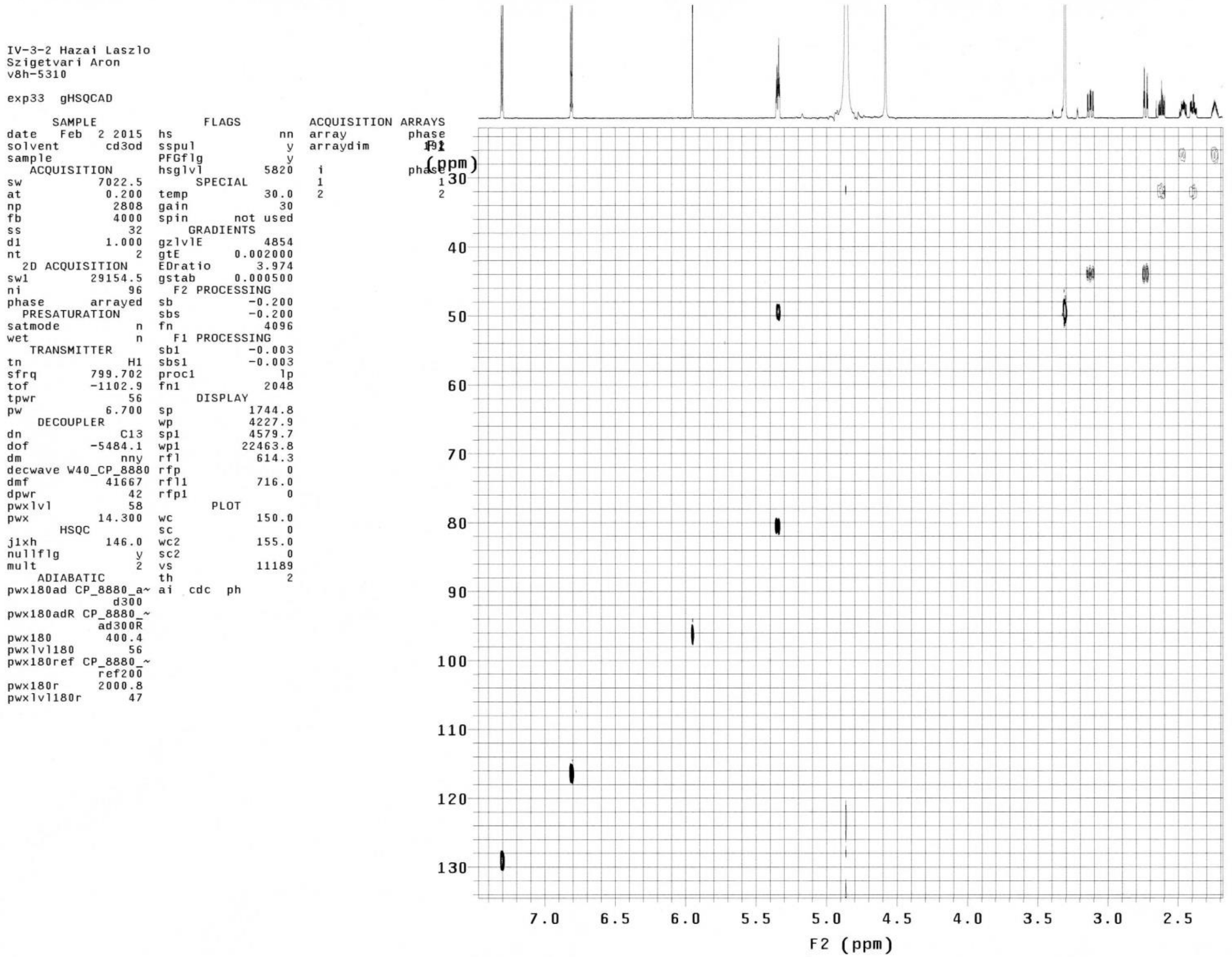

Figure S36: gHSQCad spectrum of **(±)-2a–d**.

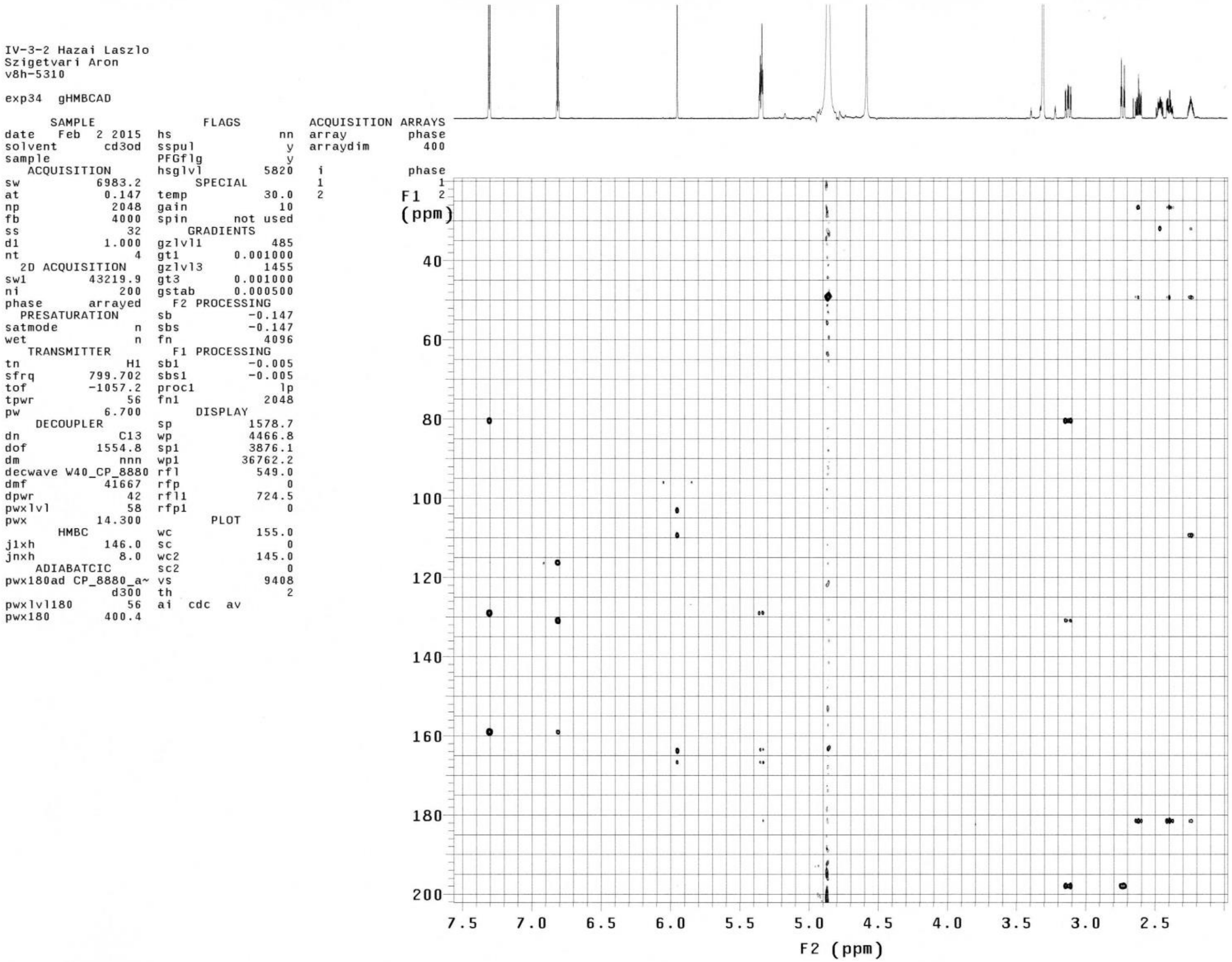

Figure S37: gHMBCad spectrum of **(±)-2a–d**.

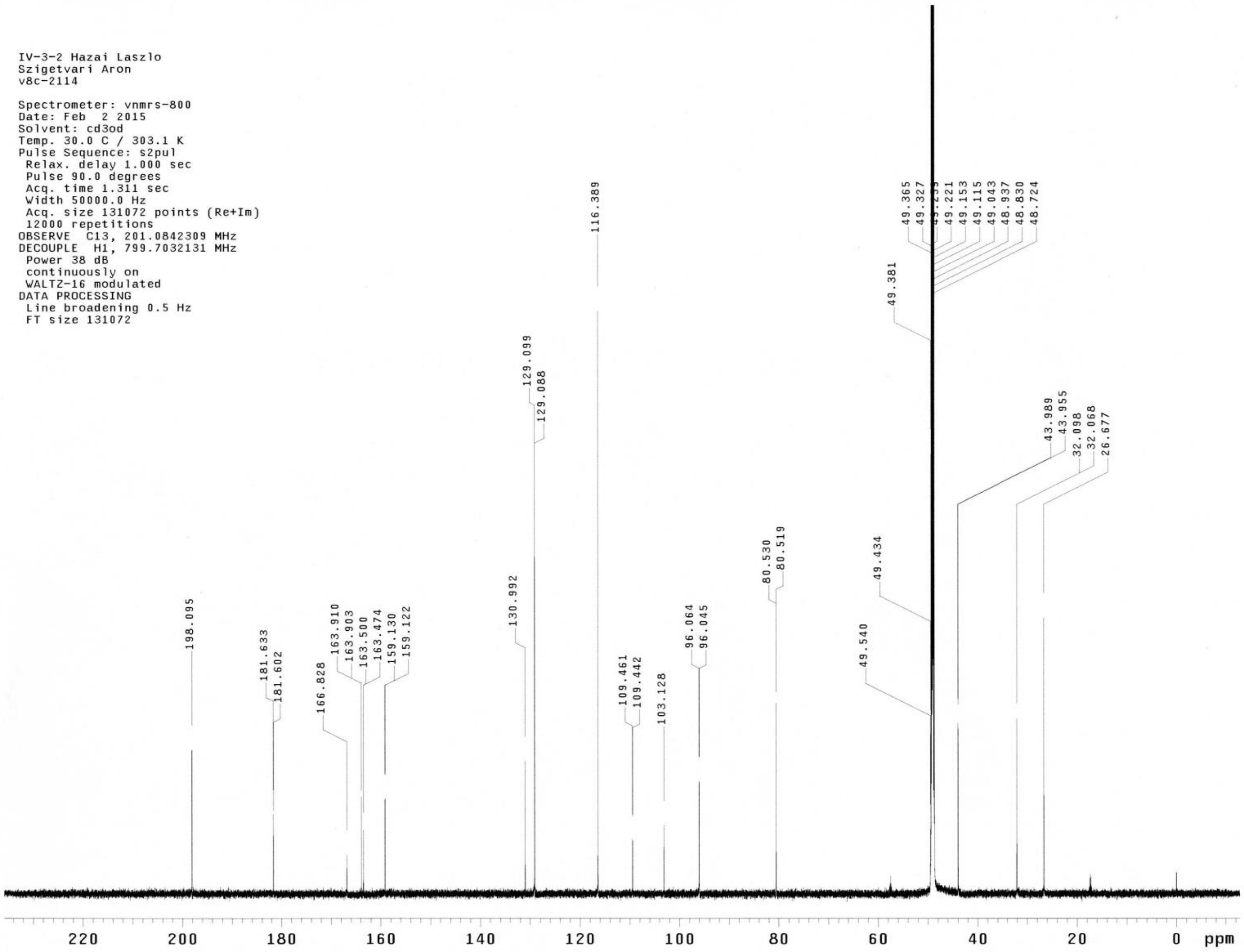

Figure S38: ^{13}C NMR spectrum of **(±)-2a–d**.
